# A Behavioral Receptive Field for Ocular Following in Monkeys: Spatial Summation and Its Spatial Frequency Tuning

**DOI:** 10.1523/ENEURO.0374-21.2022

**Published:** 2022-07-11

**Authors:** Frédéric V. Barthélemy, Jérome Fleuriet, Laurent U. Perrinet, Guillaume S. Masson

**Affiliations:** 1 Institut de Neurosciences de la Timone, UMR7289, CNRS/Aix-Marseille Université, 13385 Marseille, France; 2 Assistance Publique-Hôpitaux de Paris, Intensive Care Unit, Raymond Poincaré Hospital, 92380 Garches, France

**Keywords:** center–surround interactions, monkey, ocular following, spatial frequency, tracking eye movements, visual motion integration

## Abstract

In human and nonhuman primates, reflexive tracking eye movements can be initiated at very short latency in response to a rapid shift of the image. Previous studies in humans have shown that only a part of the central visual field is optimal for driving ocular following responses. Herein, we have investigated spatial summation of motion information, across a wide range of spatial frequencies and speeds of drifting gratings by recording short-latency ocular following responses in macaque monkeys. We show that the optimal stimulus size for driving ocular responses cover a small (diameter, <20°), central part of the visual field that shrinks with higher spatial frequency. This signature of linear motion integration remains invariant with speed and temporal frequency. For low and medium spatial frequencies, we found a strong suppressive influence from surround motion, evidenced by a decrease of response amplitude for stimulus sizes larger than optimal. Such suppression disappears with gratings at high frequencies. The contribution of peripheral motion was investigated by presenting grating annuli of increasing eccentricity. We observed an exponential decay of response amplitude with grating eccentricity, the decrease being faster for higher spatial frequencies. Weaker surround suppression can thus be explained by sparser eccentric inputs at high frequencies. A difference-of-Gaussians model best renders the antagonistic contributions of peripheral and central motions. Its best-fit parameters coincide with several, well known spatial properties of area MT neuronal populations. These results describe the mechanism by which central motion information is automatically integrated in a context-dependent manner to drive ocular responses.

## Visual Abstract

**Figure:**
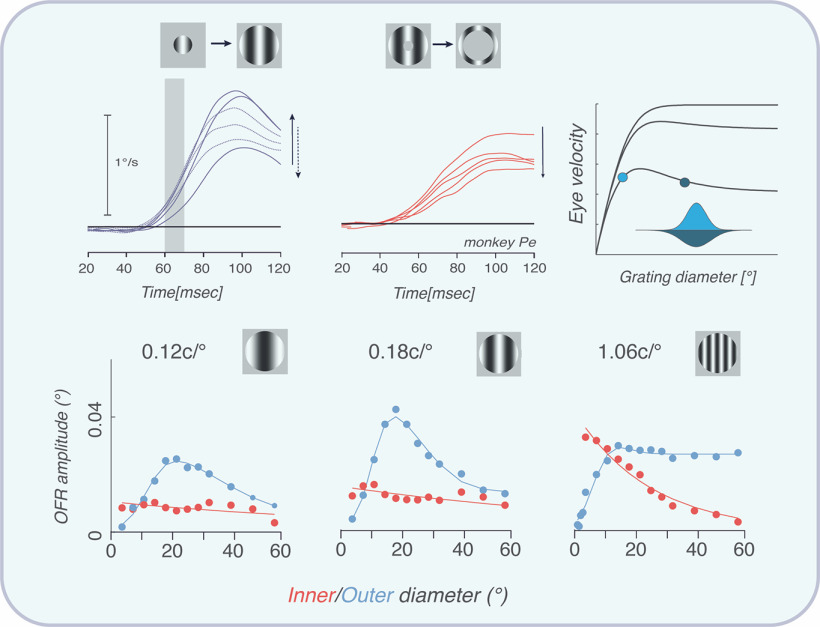

## Significance Statement

Ocular following is driven by visual motion at ultrashort latency in both humans and monkeys. Its dynamics reflect the properties of low-level motion integration. Here, we show that a strong center–surround suppression mechanism modulates initial eye velocity. Its spatial properties are dependent on the spatial frequency of visual inputs but are insensitive to either its temporal frequency or speed. These properties are best described with a difference-of-Gaussians model of spatial integration. The model parameters reflect many spatial characteristics of motion-sensitive neuronal populations in monkey area MT. Our results further outline the computational properties of the behavioral receptive field underpinning automatic, context-dependent motion integration.

## Introduction

Integrating motion signals across the visual space is essential for accurately estimating both the speed and direction of moving objects. Spatial summation plays a major role in improving the sensitivity of motion detection mechanisms (Dakin et al., 2005). The classic view suggests that motion signals are pooled over an optimal portion of visual space corresponding to the size of linear motion integration mechanisms. For neurons, such an optimal size covers the central, driving part of the receptive field. For behaviors, it is defined as the size that provides the best level of performance. With stimulus sizes beyond this limit, saturation or even a reduction in response strength or sensitivity is often observed, signaling center–surround interactions (for review, see Allman et al., 1985; Born and Bradley, 2005; Tadin, 2015). Saturation indicates a weak contribution of peripheral inputs whereas reduction unveils surround suppressive influences. Overall, the spatial properties of both the pooling mechanism and its contextual modulation determine the characteristics of an automatic, adaptive motion integration/segmentation mechanism (Tadin et al., 2019).

In both humans and monkeys, properties of visual motion integration can be probed behaviorally by measuring ocular following eye movements. Ocular following responses (OFRs) are driven by visual motion at ultrashort latencies [∼55 ms in monkeys (Miles et al., 1986); ∼90 ms in humans (Gellman et al., 1990)] and the tuning characteristics of their initial, open-loop phase reflect many of the properties of early motion detectors (for review, see Masson, 2004; Masson and Perrinet, 2012). Moreover, in macaque monkeys, ocular following properties are strongly correlated with population tuning of neuronal responses recorded in MT and MST cortical areas (Kawano et al., 1994; Miura et al., 2014). In their seminal study, Miles et al. (1986) first showed that reflexive tracking responses in monkeys were not driven by the en masse translation of the visual scene. On the contrary, when using very low spatial frequency (SF) drifting gratings, they reported an optimal size of ∼40° diameter in the central part of the visual field. Beyond it, the initial eye acceleration saturated. More recently, several groups have shown that, in humans, motion signals are pooled within the central ∼20° of the visual field for driving ocular following at very short latency (Barthélemy et al., 2006; Sheliga et al., 2008a,b 2012, 2013, 2015; Quaia et al., 2012). This optimal portion of the visual field defines the central, driving portion of an automatic, population-based mechanism underlying motion integration (Barthélemy et al., 2006). Whether such an integrating mechanism is linear or sublinear would depend on stimulus properties, in particular its orientation and spatial frequency (Barthélemy et al., 2006; Quaia et al., 2012; Sheliga et al., 2012, 2015).

There is also some evidence for nonlinear interactions shaping spatial integration of motion signals. Using center–surround stimuli, Barthélemy et al. (2006) reported that in human ocular following, a peripheral grating surrounding the central grating suppresses ocular following responses. Such suppression is dependent on the relative orientation and contrast between central and surround inputs. Using pairs of thin motion stripes that can be positioned at different eccentricities, Sheliga et al. (2008a,b, 2012) mapped suppressive interactions between center and peripheral motions (Sheliga et al., 2008a,b, 2012; Quaia et al., 2012). Their results support the view that peripheral and central motion signals interact such that spatial summation for reflexive tracking initiation stems from a sublinear integration of central inputs, modulated by suppressive inputs from the near periphery acting through a divisive normalization. Such modulation is dependent on several statistical properties such as the orientation, contrast, or distances of these flankers.

Barthélemy et al. (2006) called this integration mechanism a behavioral receptive field (bRF) defined as a computational unit underlying automatic, low-level motion integration (Masson and Perrinet, 2012). The bRF reflects a cascade of neural mechanisms acting at population level and implementing linear and nonlinear computations such as local and global normalization (Perrinet and Masson, 2007; Quaia et al., 2012). The question of which neural stages, from the retina to extrastriate cortical areas, constrain these computational steps remains open. An essential piece of information in mapping behavior to neural level has always been to first characterize these properties at the behavioral level in macaque monkeys. Visual signals driving ocular tracking responses are ultimately extracted from population activities in areas MT and MST (Kawano et al., 1994; Takemura et al., 2007; Miura et al., 2014). Spatial and temporal properties of these retinotopic neural networks can be related to both spatial summation and normalization mechanisms shaping the bRF dynamics. Here, we unveil several properties of spatial summation and their dependencies on stimulus spatial frequency and position within the visual field.

## Materials and Methods

### Animals

Two male, adult rhesus monkeys (No, Pe) were used in the present study. On each animal, a surgical procedure was performed under isoflurane anesthesia. A Teflon-coated three-turn magnetic search coil (catalog #AS 632, Cooner Wire) was sutured to the sclera under the conjunctiva of one eye (Fuchs and Robinson, 1966; Judge et al., 1980) to measure the orientation of gaze (eye-in-space) with the electromagnetic search-coil technique (Skalar; Robinson, 1963). Lead wires were passed under the skin to a connector located on the top of the skull. During the same procedure, a head restraint fixation was positioned on the top front center of the skull and secured with bone cement (Palacos, Smith and Nephew) layered about stainless screws attached to the skull. Training was initiated after full recovery. Animals were paired for housing. Daily experiments were conducted in the morning. All animal procedures were performed in accordance with the regulations of the Aix-Marseille Université animal care committee.

### Eye movements recording and behavioral paradigm

Data acquisition, online control of behavior, and stimulus triggering were controlled by a PC using the REX 7.7 software package with the real-time QNX operating system (Hays et al., 1982). Voltage signals separately encoding horizontal and vertical positions of the right eye were low-pass filtered (6 poles ; DC, 180 Hz; Bessel) and sampled at 1 kHz, with a resolution of 16 bits. Monkey were seated in a fiberglass chair, with head restrained, and faced a large (70° × 70°) vertical screen at a viewing distance of 1 m. Visual stimuli were back-projected using a high-resolution BARCO 809s video projector (resolution, 1280 × 1024 at 96 Hz; frame duration, 10.4 ms). Visual stimuli were precomputed movies, generated using the HIPS libraries (Landy et al., 1984) and stored in the memory of a SGI Fuel workstation. The visual workstation and experimental PC communicated through a serial port. Synchronization between the two computers has been fully described previously (Masson et al., 2000).

We used a simplified version of the behavioral paradigm that has been extensively described previously in monkeys (Miles et al., 1986; Masson et al., 1997) and humans (Gellman et al., 1990; Masson and Castet, 2002). Trials started with a uniform background of mean luminance (22.5 cd/m^2^), and a target spot produced by a light-emitting diode back-projected onto the screen, at the center. Monkeys were required to fixate this spot for a time interval of random duration, after which the spot disappeared. After a 50 ms gap, the motion stimulus was presented for 220 ms (22 frames) before the screen was blanked, ending the trial. In the different experiments, all conditions were fully randomized and interleaved with “catch trials” in which no stimuli were presented after the extinction of the fixation. Monkeys were rewarded by one drop of water only if fixation was maintained within 1° of both horizontal and vertical target positions and no saccades were made during both fixation and stimulus motion periods. Eye positions were monitored online by the REX software using electronic windows.

### Visual stimuli and experimental conditions

Motion stimuli were always vertical sinusoidal luminance gratings drifting either rightward or leftward. Display luminance levels were calibrated and linearized by means of a lookup table. In all experiments, drifting directions were fully randomized across trials such that the next motion stimulus was unpredictable and day-to-day fluctuations in response amplitude were evenly randomized across all conditions. Still, we analyzed ocular following responses to either direction separately. Two types of stimulus spatial shapes were used where sinewave luminance gratings were presented either within a disk or a ring. For disk-like conditions, motion stimuli were presented within a circular aperture, centered on the initial fixation point location. To probe the spatial summation of ocular following, we varied the diameter of the aperture and thus the motion stimulus surface area. For ring-like stimuli, we generated a moving grating within a circular aperture of very large diameter (60°) but covered its central part with a uniform disk of mean luminance, thus creating a ring of grating motion. By varying the diameter of the inner disk, we can probe the contribution of peripheral motion. In the different experiments, the grating spatial and temporal frequencies were also manipulated. Overall, we ran four different experiments in our two animals.

#### Experiment 1: measuring spatial summation at optimal spatial frequency and speed

In a first series of experiments, ring and disk-like stimuli were presented at a spatial frequency of 0.36 cycles/° (cpd) and a speed of 30°/s, corresponding to a 10.8 Hz temporal frequency. These parameters have been previously reported to be optimal to drive short-latency ocular following responses in macaque monkeys (Miles et al., 1986). Disk and ring-like stimuli were interleaved. The diameter of the disk stimulus was systematically varied from 3° to 55° in 12 steps (3.55, 7.1, 10.65, 14.20,17.75, 21.3, 24.85, 28.4, 31.95, 39.05, 46.15, and 53.25°). Thus, the surface of the motion stimulus in central vision increased from ∼20 to 4454°^2^. The different ring sizes were set by increasing the diameter of the mean luminance inner disk with the same 12 steps (diameter, 3.55–53.25°). Thus, the surface of the ring-like motion stimulus decreased from ∼5635 to 1200°^2^. Note that the surface for the most eccentric ring-like stimulus (inner diameter, 53.25°) was approximately identical to the surface of the 28.4° diameter disk-like stimulus.

#### Experiment 2: effect of speed/temporal frequency on spatial summation

In a second experiment, the same set of 12 disk diameters was presented at the same spatial frequency (0.36 cpd) but at three different speeds (15, 30, and 45°/s), corresponding to temporal frequencies of 5.4, 10.8, and 16.2 Hz. All speeds and directions (leftward/rightward) were interleaved.

#### Experiment 3: effect of grating spatial frequency on spatial summation

In the third experiment, we measured spatial summation across a broad range of grating spatial frequencies. Six different spatial frequencies were tested (0.12, 0.18, 0.36, 0.72, 1.06, and 1.41 cpd) spanning ∼3.6 octaves within the optimal spatial frequency range for ocular following responses in macaque monkeys (Miles et al., 1986). As speed, but not temporal frequency, is known to impact OFR amplitude in monkeys (Miles et al., 1986), we fixed the grating motion speed at a constant value (30°/s) by varying its temporal frequency. The six spatial frequencies were thus presented at six temporal frequencies, ranging from 3.6 to 42.3 Hz. The same set of 12 different disk diameters was tested. Throughout this experiment, we also probed the contribution of peripheral motion signals across the same grating motion conditions (30°/s, left/rightward) and the same range of spatial frequencies (0.12–1.41 cpd). Ring stimuli of the same 12 different inner diameters were interleaved with the disk conditions.

In one monkey (Pe), we found that our smallest stimulus diameter (3.6°) was still too large to precisely map the rising phase of the spatial summation function for the three highest spatial frequencies (see Fig. 5, main plots). We therefore ran an additional experiment for the two animals with a set of smaller diameters, ranging from 1° to 7°. Notice that this range was overlapping with the original one (3.55–53.5°), enabling comparison of spatial summation functions across the two datasets. This complementary experiment was run with the same grating motion conditions (30°/s, left/rightward motion direction) and the three highest spatial frequencies (0.72, 1.06, and 1.41 cpd).

#### Experiment 4: testing the effects of eccentric line endings

Masking different parts of a motion stimulus can have different consequences that must be taken into consideration when mapping the spatial summation function. First, with the ring stimulus, increasing the inner disk diameter both reduced the overall surface area of the stimulus and increased the mean eccentricity of motion inputs. For the largest inner diameter, this annulus-like aperture extended from 26.6° to 30° of eccentricity, leaving only a thin ring of 3.4° in width. Second, introducing a mean luminance disk at the center of the grating stimulus generated line-ending motion features at the intersection between the grating and aperture luminance profiles. These line-ending features translate along the edge of the circular aperture, with a mean velocity equal to that of the grating motion. Third, notice that such line-ending motions were also present along the external border of the disk stimulus, although with very large eccentricity (>30°). These local motion signals are known to impact late, but not early, phases of ocular following responses in both humans (Masson et al., 2000) and monkeys (Barthélemy et al., 2010) and might affect spatial summation (Sheliga et al., 2015). To check their potential contribution to the dynamics of spatial summation, and its dependency on eccentricity, we ran a final, control experiment. A thin (10° wide) ring of mean luminance was superimposed to the full, 60° diameter drifting grating. The eccentricity of this ring mask was varied using the same values as the inner diameter of the ring motion stimulus. Spatial frequency of the moving grating was set at the optimal frequency for each monkey. Grating speed was set at 30°/s, and the grating drifted either to the left or to the right. Again, all conditions were interleaved.

### Data analysis

Experiments were organized into several daily recording sessions of ∼2 h duration, usually collecting ∼150–200 trials for each condition. The signal-to-noise ratio necessary to adequately resolve the responses was then achieved through averaging. The data from all sessions were pooled and analyzed offline, as described previously by Masson et al. (2000). To eliminate possible contamination of the eye velocity profiles from spurious slow ocular drifts, all data shown here have a fixation-only (blank stimulus) condition subtracted.

Quantitative analysis was performed by measuring, for each trial, the changes in horizontal eye position over several successive 10 or 20 ms time bins spanning from 60 to 120 ms after stimulus onset. This overall 60–120 ms period thus analyzed corresponds to the open-loop period of the visuomotor transformation, that is when the displacement of the eye has not yet affected the visual processing of the retinal image given the visuomotor delay (∼60 ms; Miles et al., 1986). Moreover, using several successive time windows, time-locked with stimulus onset allows mapping of the temporal dynamics of responses to complex motion inputs (Masson and Castet, 2002). Spatial summation functions were computed for each condition by plotting mean response amplitudes against the diameter of the disk stimulus. The relative contribution of peripheral motion was then estimated by plotting mean response amplitudes against the size of the inner diameter of the ring stimulus. These plots were computed for each time window to illustrate the temporal dynamics of such motion integration. Finally, we checked the effects of stimulus size on early time windows by analyzing the earliest peak acceleration of the ocular following responses. Mean eye velocity profiles were computed. The blank-only condition was subtracted, and the corrected eye velocity profiles were differentiated to obtain mean eye acceleration profiles. The first maximum of eye acceleration was extracted, together with its time of occurrence, and plotted relative to stimulus size. Since results with eye acceleration were similar to those with change in eye positions, we do not report them systematically.

### Modeling spatial summation function

Visual integration is classically best described by the spatial parameters of some spatial summation function, and in particular the extent of the excitatory and inhibitory mechanisms and the contribution of peripheral motion. We decided to compare two approaches, similar to that of Cavanaugh et al. (2002) for V1 neurons. First, a model-free method was used to extract these properties directly from the observed relationships between response amplitudes and disk or ring diameters. Specific response amplitude criteria were defined to extract each spatial parameter of motion integration. For each parameter, its value was set by the data point that is the closest from the corresponding amplitude criterion. Such model-free extraction of characteristic parameters is important as some properties such as center extent or surround extent (SExt) could be estimated from different spatial summation models (Cavanaugh et al., 2002). However, their precision is constrained by our experimental sampling. Therefore, a second model-based method was used to better extract the spatial properties of visual motion integration. We fitted the data with two descriptive functions using the Levenberg–Marquardt algorithm (MATLAB, MathWorks). The first model estimates the properties of spatial summation from a difference-of-Gaussians (DoG) model as already used by others for human ocular following (Barthélemy et al., 2006; Perrinet and Masson, 2007; Sheliga et al., 2013). The second model characterizes the contribution of peripheral motion using an exponential decay function. These two mathematical models will be described at the end of the Results section. For both models, the goodness of fit was estimated by computing a normalized $\chi_{N}^{2}$ value (Cavanaugh et al., 2002).

## Results

Below, we will first describe the effects of stimulus size on short-latency ocular following responses when presented with a low spatial frequency, drifting grating. We will also describe, for this same spatial frequency, the contribution of peripheral motion when using ring-like stimuli. Next, we will report how the properties of spatial summation varied with the spatial frequency of the drifting stimulus, while keeping temporal frequency constant. Properties of both spatial summation and eccentricity dependency functions for ocular responses will be estimated from two independent methods. First, we extracted several characteristic points directly from the observed data of each monkey, as defined by Cavanaugh et al. (2002). Second, individual spatial summation functions were fitted with a DoG model to better estimate the properties of the center–surround mechanisms that define a behavioral receptive field in monkeys, as previously done in humans (Barthélemy et al., 2006; Perrinet and Masson, 2012; Sheliga et al., 2013). The contribution of eccentricity inputs at different spatial frequencies will be estimated by fitting an exponential decay function. These model-based statistical estimates can then be compared with the known properties of neuronal spatial summation at various stages along the macaque cortical motion pathway.

### Spatial summation function at low spatial frequency

**Figure 1.**
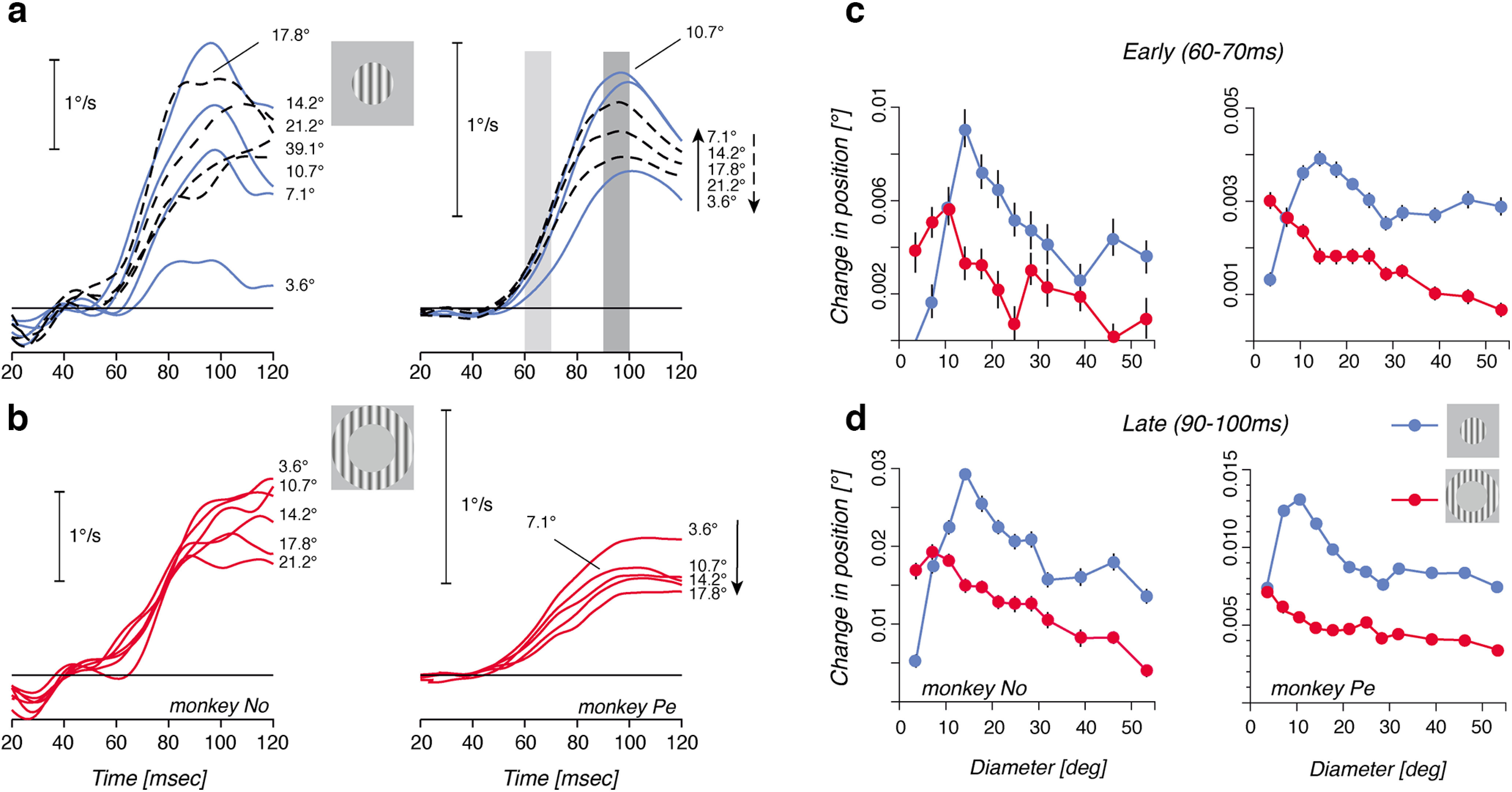
Ocular following responses are modulated by the size of the motion stimulus. ***a***, Mean eye velocity profiles of ocular following responses triggered by a vertical, rightward drifting grating at constant spatial and temporal frequencies (experiment 1). The motion stimulus was presented behind a circular aperture of increasing diameters. As this diameter increased from 3.6° to 14.2°, the initial eye velocity increased (continuous lines). For larger and larger stimuli, initial eye velocity gradually decreased (broken lines). Right-hand numbers are grating patch diameters. ***b***, Mean eye velocity profiles of ocular following driven by the same grating motion presented within a large (diameter, >50°) patch. The central portion of the stimulus was covered by a mean luminance patch of increasing diameter, as indicated by the numbers at the right-end of each curve. Data are illustrated for the two monkeys, No and Pe. Notice that the small initial dip in eye velocity seen at ∼20 ms for monkey No resulted from the subtraction of the mean velocity profile of the catch trials, where a tiny, idiosyncratic deflection was present at the end of fixation. Such an artifact occurred at very short latency (<20 ms) and therefore did not impact our quantitative analysis of response amplitudes. ***c***, ***d***, Mean change in position during the early (***c***; 60–70 ms) and late (***d***; 90–100 ms) time windows, plotted as a function of the grating patch diameter (blue curves) or inner mean luminance patch diameter (red curves). Data are the mean ± SE, over >150 trials for each condition. Left-hand and right-hand columns are data from monkeys No and Pe, respectively.

We first investigated the effects of stimulus size on the amplitude of ocular following responses when using drifting gratings of 0.36 cpd (experiment 1). Figure 1*a* illustrates, in two monkeys, ocular following responses elicited by rightward motion of a vertical grating presented behind circular apertures of diameter increasing from 3.6° to 53°. Responses were initiated at a latency of ∼55 ms, albeit a slightly longer latency (∼60 ms) was observed with the smallest stimulus. Overall, no systematic change in response latency was observed when increasing stimulus size >3.5°. By contrast, increasing stimulus size from 3.5° to 21° diameter resulted in a sharp increase in initial eye velocity (continuous lines), and thus larger responses, in both animals. Maximal responses were observed with stimulus sizes of ∼10-15° diameter. Further increasing grating size beyond such optimal diameter resulted in a large decrease in the initial eye velocity (dashed curves). Overall, for very large stimuli, eye velocity at the end of the open-loop period converged to some asymptotic value at ∼75% of the maximum eye speed reached with the optimal stimulus size.

For each condition, and across all the ∼150 trials recorded for each direction and animal, we quantified response amplitudes by measuring the changes in horizontal eye position over 5 successive 10 ms time windows. Blue symbols in Figure 1, *c* and *d*, illustrate the relationship between response amplitude and the outer diameter of disk-like stimuli immediately after tracking initiation (60–70 ms time window, top row) and right before the closing of the visuomotor loop (90–100 ms time window, bottom row). For both monkeys, response amplitudes peaked for a stimulus diameter of ∼15°. For larger diameters between 15° and 30°, both initial and late responses decreased, reaching a plateau for diameters beyond ∼30°. No significant changes in the shape of the curves were observed between early and late measurements, except a better signal-to-noise ratio for the late time window.

In the same experiment, we also estimated the contribution of peripheral motion by presenting a very large grating patch (diameter, 60°) while covering a larger and larger portion of the central visual field with a circular mean luminance disk of increasing size. We called this stimulus a ring in which the inner diameter was manipulated. Figure 1*b* illustrates ocular following responses to ring-like stimuli. All responses have a nearly similar ultrashort latency of ∼55 ms after stimulus onset. However, the initial eye acceleration was strongly modulated: responses decreased as more and more of the central stimulus was covered. Responses amplitude is thus scaled by the grating surface. Moreover, the smallest, but still consistent, responses were observed with the largest inner diameters (up to 53.25°). This result suggests that ocular following can be driven by visual motion inputs located far in the periphery (up to ±30° in our experimental conditions) and covering only a small fraction of the visual field. Red symbols in Figure 1, *c* and *d*, plot early and late response amplitudes against ring inner diameter, respectively. In the two monkeys, we found a monotonic relationship between ocular following amplitude and the inner diameter of the ring. The small increase seen in monkey No when small-diameter disks of mean luminance were introduced (inner ring diameter, <10°) was not systematically observed with the other monkey or with the other tested conditions (see below).

### Quantifying the properties of spatial summation for ocular following

**Figure 2.**
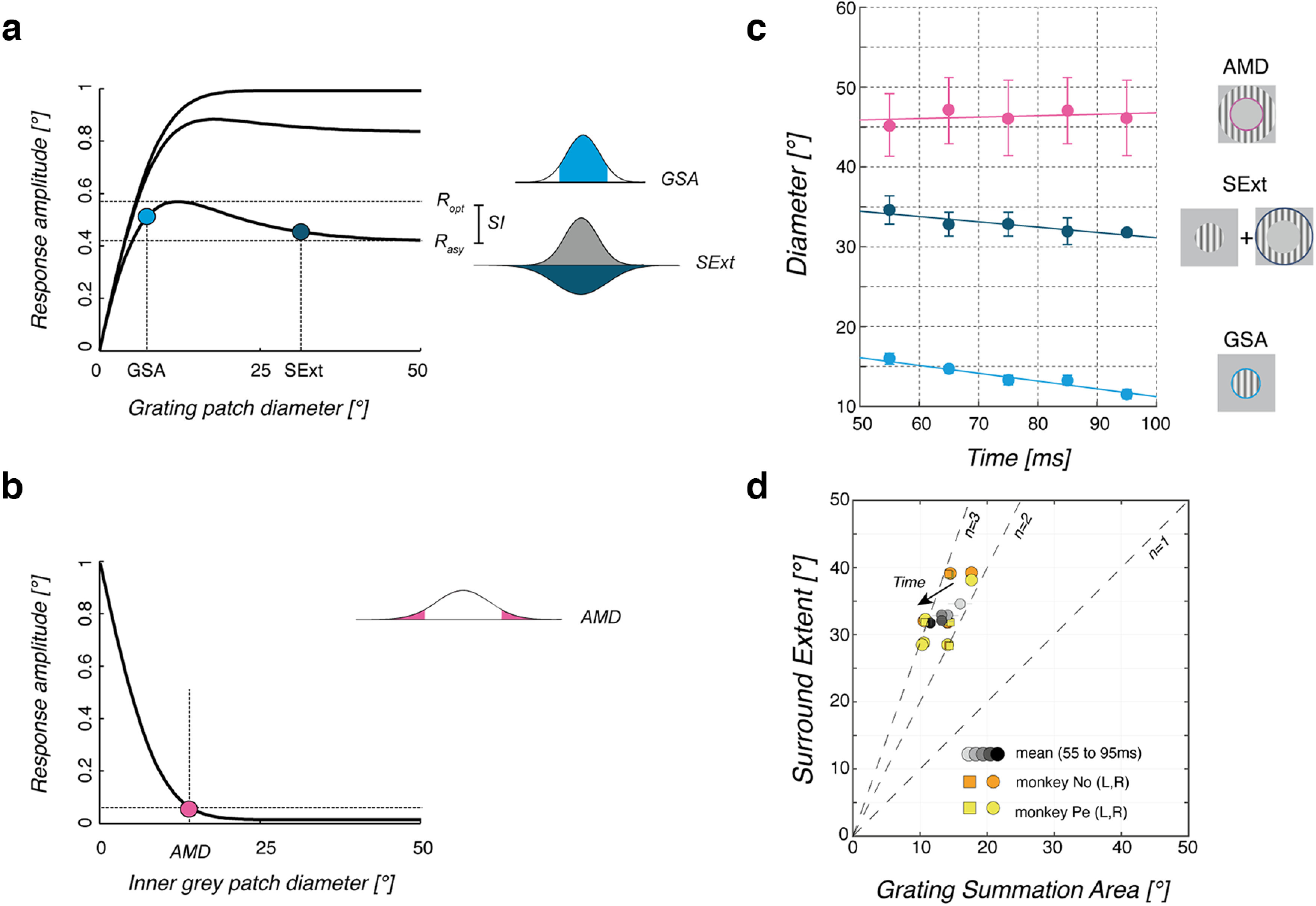
Characteristics of spatial summation for ocular following. ***a***, Three different shapes of a spatial summation function are plotted to illustrate how different characteristic parameters can be extracted to describe relationships between stimulus size and response strength. Top and bottom horizontal lines illustrate the maximum (*R*_opt_) and plateau (*R*_asy_) OFR amplitude levels. The GSA is defined as the first smallest stimulus diameter for which response amplitude reached 95% of the maximum level (light blue symbol). They correspond to a stimulus size covering 95% of a central driving field, schematized as a Gaussian profile drawn over the visual field. SExt is the first stimulus diameter yielding to a response amplitude within 5% of the plateau level (*R*_asy_). This would correspond to the size of the stimulus covering the driving field but also a large portion of a peripheral, inhibitory surround field. The SSI is the ratio between the plateau and maximum levels of the raw data. ***b***, Theoretical relationship between response amplitude and the size of the central, occluding mean luminance patch of the ring stimulus. The AMD is the first inner diameter for which the response amplitude reached 5% of the smallest response, as observed with the largest mean luminance patch. This value characterizes the portion of the visual field from which no significant motion signals can be used to drive ocular following. GSA, SExt, SSI, and AMD were computed for each monkey and each motion direction or speed. ***c***, GSA, SExt, and AMD mean values (mean ±SE across monkeys and directions) are plotted for five different time windows covering the entire open-loop period of the ocular responses. ***d***, GSA and SExt are plotted one against each other. Dotted lines correspond to a ratio of 1, 2, or 3 between the two spatial parameters. Data are both individual estimates (each monkey and direction) and mean values (error bars are the SE across monkeys and directions) for each of the five time windows. To illustrate the temporal dynamics, mean values for successive time windows are plotted with increasing gray levels.

Similar to neuronal visual motion integration, expanding a patch of grating first increases the ocular response amplitude up to a maximum, defining the region of summation. However, for larger patches, response amplitude decreases, revealing suppression as the stimulus engages the inhibitory surround. Reconstructing the spatial structure of the excitatory center and inhibitory surround mechanisms of motion integration for eye movements faces two challenges. First, one needs to extract the spatial parameters of center and surround from the size–amplitude relationships. Second, the surround is not silent, as observed for the neuronal receptive fields, as peripheral motion can still drive ocular responses. We need therefore to estimate the weight of these peripheral inputs on their own. Our first analysis step was to extract the spatial characteristics of center and surround mechanisms directly from our data, as model-free parameters that can be defined from several response amplitude thresholds. To do so, we used the same spatial measurements as defined by Cavanaugh et al. (2002) for characterizing center–surround spatial organization of V1 neurons (Fig. 2*a*,*b*). These estimates assume that center and surround mechanisms are best depicted by pooling of inputs of two Gaussians, but without any other assumptions about their interaction mechanisms as in model-based approaches. They were extracted for each monkey and grating motion direction by taking the observed data point that matched the definition of the parameter, with no fitting or interpolation.

The grating summation area (GSA) was defined as the diameter of the smallest stimulus that elicited at least 95% of the tracking maximum response (Fig. 2*a*, light blue). It indicates the optimal diameter of the excitatory field. For the earliest ocular following, the mean ± SD (across monkeys and directions) GSA was of 15.9 ± 2.1°. For the latest time window, the mean GSA was significantly smaller (11.5 ± 1.7°, *t*_(3)_ = 3.96, *p* = 0.028). Overall, GSA slowly reduced over the entire open-loop period of ocular following (Fig. 2*c*, light blue symbols), as shown by the weak but significant decreasing linear regression between GSA and time, across monkeys and directions (slope, −0.1; intercept, 21°; *r*^2^ = 0.46, df * *= 18, *p* < 0.05). Stimulating a larger portion of the visual field always caused a significant reduction in initial eye velocity, illustrated by the lower response amplitude for stimulus diameters beyond the GSA. Such reduction increased as the stimulus expanded into the near periphery until it reached a plateau for very large diameters. We defined the SExt as the diameter of the smallest stimulus for which initial eye velocity was within 5% of the asymptotic value observed with the largest grating diameter (Fig. 2*a*, dark blue). SExt corresponds to the optimal diameter of the inhibitory field. The mean SExt (±SD across monkeys and directions) was of 34.6 ± 5.6°. Figure 2*c* shows that SExt (dark blue symbols) remained constant over the open-loop period (linear regression: slope = −0.7, intercept = 37.8, *r*^2^ = 0.05, df* *=* *18; NS). Moreover, as illustrated in Figure 2*d*, we observed that the SExt was approximately two times larger than GSA, and their ratio slowly but significantly increased over time (e.g., mean ± SD across monkeys and directions: 2.2 ± 0.11 and 2.8 ± 0.36 for the earliest and latest time windows; *t*_(7)_ = 0.49, *p* < 0.01). We found a weak linear correlation between time and SExt/GSA ratio (linear regression: slope = 0.014, intercept = 1.37, *r*^2^ = 0.36, df* *=* *18, *p <* 0.1). Altogether, these results show that, over time, the central area of integration shrinks but the surround remains nearly constant.

Last, we computed a surround suppression index (SSI) as the normalized reduction between the largest ocular following amplitude (*R*_opt_) and the asymptotic amplitude obtained with very large stimuli (*R*_asy_):

$$\text{SSI}=\frac{\left(R_{\text{opt}}-R_{\text{asy}}\right)}{R_{\text{opt}}}.$$

Thus, SSI estimates the surround inhibition strength. For the earliest ocular following, SSI ranged between 0.36 and 0.69 (mean ± SD, 0.51 ± 0.19, across the two monkeys). Similar values were found for the other time windows (i.e., 90–100 ms, 0.41 ± 0.16), indicating that the suppression stayed constant at nearly 50% over the open-loop period of tracking initiation (linear regression: slope = −0.01; intercept = 0.58, *r*^2^ = 0.03, df* *=* *18; NS).

To probe the contribution of peripheral motion, we analyzed the relationships between response amplitudes and the inner diameter of the grating ring. As illustrated in Figure 1, *c* and *d*, removing a larger and larger central portion of the moving grating resulted in a regular decrease in the initial eye velocity of ocular following responses. Following Cavanaugh et al. (2002), we defined the annular minimal diameter (AMD) as the inner diameter for which response amplitude first dropped to <5% of the peak response observed with the largest grating stimulus (Fig. 2*b*). Across monkeys and directions, the mean ± SD AMD values were 47.1 ± 12°, indicating that motion signals located very far in the peripheral field can elicit only small, but consistent, ocular following. However, initial eye velocity, as well as peak eye velocity, obtained with grating rings were always smaller than those obtained with grating patches. Furthermore, response amplitudes of the grating ring (inner diameter, 53°) covering a surface almost identical (∼2400°^2^) to a matched grating patch (diameter, 28°) were always smaller, indicating a stronger weight for central motion signals. Across directions and monkeys, such a mean (±SD) reduction between central and peripheral inputs was 74 ± 5% for the earliest time window (60–70 ms after stimulus onset). As illustrated in Figure 2*c* (pink symbols), AMD remained constant over the open-loop time period (linear regression: slope, 0.02; intercept, 44.9; *r*^2^ = 0.1; NS).

In summary, for a 0.36 cpd moving grating, the nonmonotonic relationship between eye velocity and stimulus diameter can be explained by an integrative center of ∼15° diameter and a suppressive surround twice this size (∼35°). Such a suppressive surround reduces response amplitude by ∼50% at the largest stimulus diameters, relative to the optimal size. Moreover, visual motion presented in the far periphery (ring inner diameter, 53.25°) can drive ocular following, albeit with a much smaller response than a central motion patch covering the same surface (disk diameter, ∼28°) with an ∼74% reduction factor between equivalent central and peripheral input. It shall, however, be recalled that GSA, Sext, and AMD parameters were defined to capture the spatial properties of visual motion integration directly and only from the tested conditions. Therefore, the resolution of each parameter estimate was constrained by our experimental sampling of disk and ring diameters. Below, we will compare these values with the spatial parameters of both center and surround mechanisms as estimated by fitting a model of spatial summation for gratings over a large range of spatial frequencies, including the one reported here (0.36 cpd).

### Spatial summation is constant over a range of speeds and temporal frequencies

Before measuring spatial summation for ocular following with gratings of different spatial frequencies, we ran a control experiment (experiment 2) where the spatial frequency of the grating was kept constant (0.36 cpd) but its temporal frequency, and therefore its speed was varied. We used two new temporal frequencies (5.4 and 16.2 Hz), interleaved with the original (10.8 Hz) condition, yielding to three different grating speeds (∼15, 30, and 45°/s) that span the optimal range for monkey ocular following (Miles et al., 1986).

**Figure 3.**
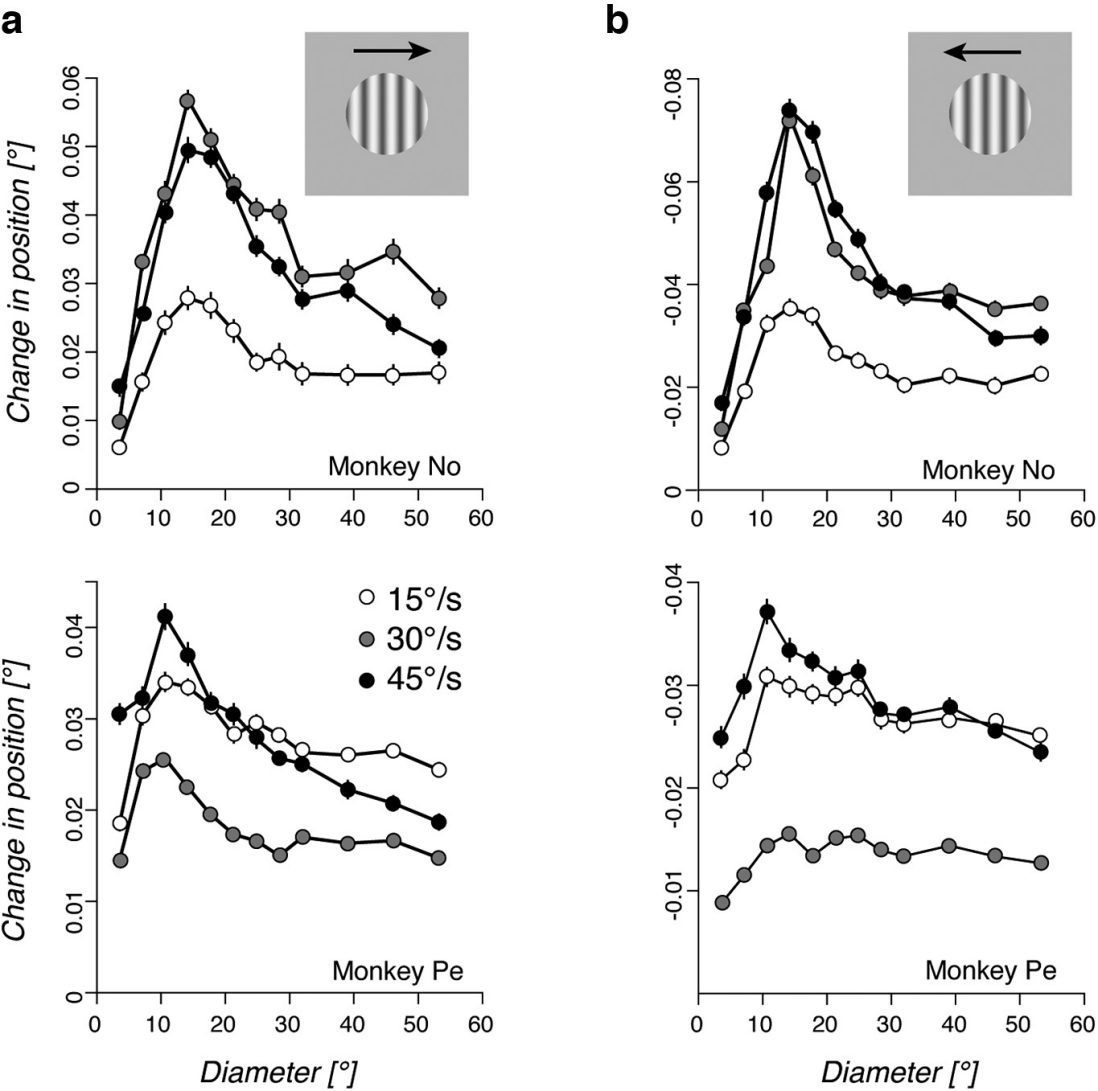
Spatial summation function is velocity independent. ***a***, ***b***, The spatial summation function of ocular following is plotted against the grating patch size for rightward (***a***) and leftward (***b***) motion directions and, in each one, for three different speeds (15, 30, and 45°/s; experiment 2). Top and bottom rows plot the results for monkey No and Pe, respectively. Data are the mean changes in position (±SE) over the 85–105 ms time window.

Overall, varying grating speed did not change the properties of spatial motion integration as illustrated in Figure 3, *a* and *b*, where the amplitude of ocular following responses over a 20 ms time window (85–105 ms) is plotted against grating patch diameter, for each monkey. Responses to rightward and leftward grating motion are plotted separately. There was an overall effect of grating speed on the global amplitude of the ocular responses, as seen by comparing the offsets of the three different curves. However, all curves peaked at a similar grating patch diameter for a given direction, yielding to nearly constant GSA across speeds (∼14°; paired *t* test, NS). Similarly, SExt values were similar across conditions (paired *t* test, *p* > 0.1). Higher speed (45°/s) elicited larger suppression, when compared with the lower speed (15°/s; SSI: 0.52 ± 0.11 and 0.30 ± 0.09, respectively; paired *t* test, *t*_(6)_ = 3.09, *p* < 0.025). It shall be noticed that, as such suppression is not scaled to the absolute response amplitude, SSI values in the two monkeys are very similar despite the fact that monkey No exhibited larger response amplitudes.

### Spatial summation at different spatial frequencies

**Figure 4.**
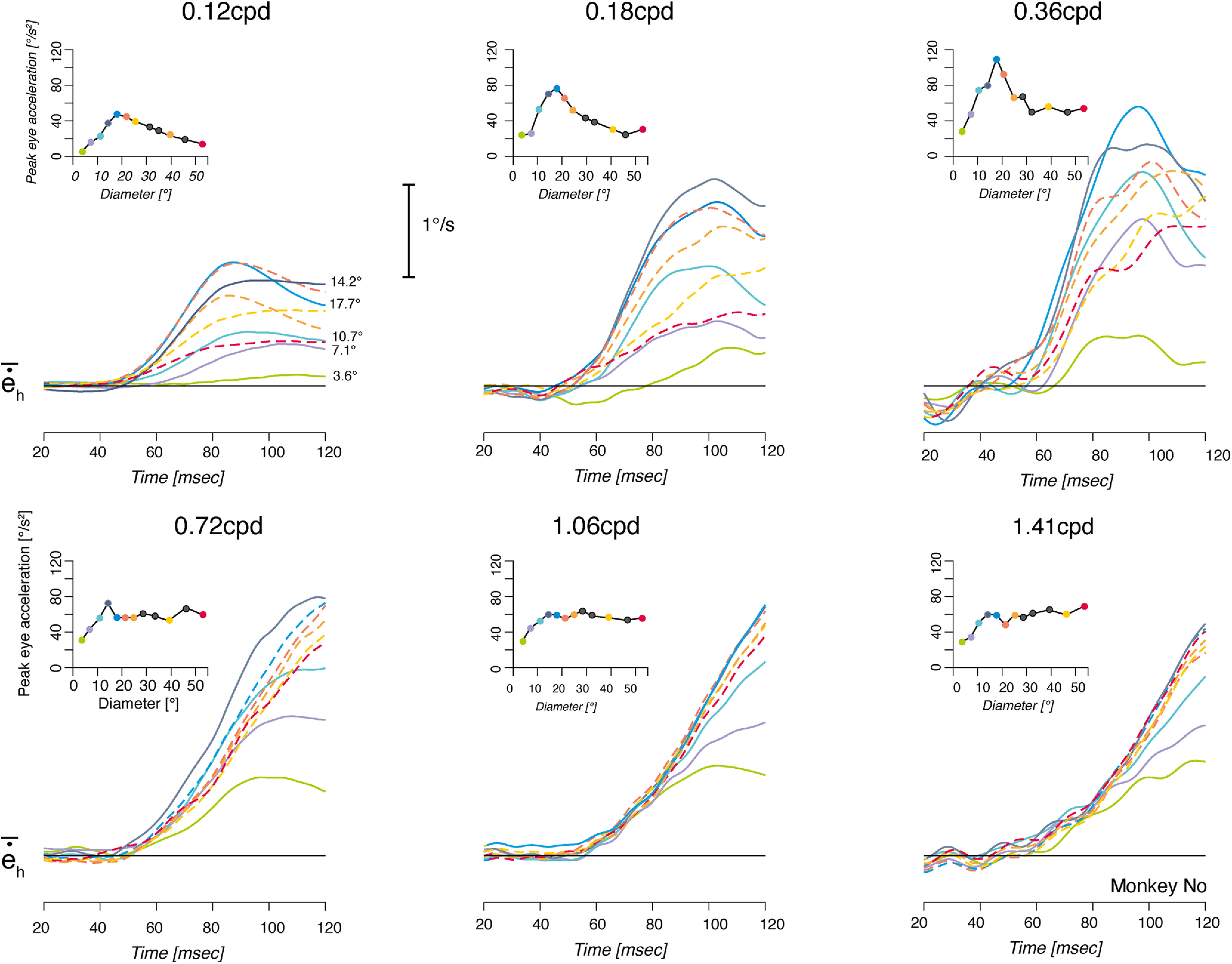
Spatial summation is spatial frequency sensitive. Mean eye velocity profiles are plotted for nine representative grating patch sizes (experiment 3; diameter, 3.55–53.25°). Broken lines illustrate decreasing eye velocity responses when increasing stimulus sizes. The mean initial eye peaks of acceleration are plotted against stimulus diameters. Corresponding dots and curves are of the same color. Each moving grating was presented at six different spatial frequencies, ranging from 0.12 to 1.41 cpd in Monkey No.

In the next experiment (experiment 3), we systematically varied the spatial frequency of the drifting grating to investigate how it affects spatial summation for ocular following. Figure 4 illustrates the eye velocity profiles observed for different stimulus diameters in monkey No, at six different grating spatial frequencies ranging from 0.12 to 1.41 cpd. Notice that the third set of curves of the top panel (0.36 cpd) corresponds to the data already plotted in Figure 1*a*. Continuous lines are responses of increasing initial eye velocity when enlarging stimulus size. Broken lines illustrate the decreasing responses observed when further widening the grating patch. At low spatial frequency (Fig. 4, top panels, 0.12–0.36 cpd), a biphasic pattern of initial eye velocity was observed, with a strong suppression of response for stimuli larger than the optimal size, as reported above. However, such surround suppression became weaker for the intermediate spatial frequency (0.72 cpd) and had largely disappeared at the two highest spatial frequencies (1.06 and 1.41 cpd; Fig. 4, bottom panels). Thus, for these last two grating spatial frequencies, response velocity simply saturated for large stimuli. This is further illustrated by the inset plots in Figure 4, where the first peak of eye acceleration is plotted again stimulus diameter. Each color circle in the inset plots in Figure 4 corresponds to a velocity profile and thus a given spatial frequency. Initial eye acceleration first linearly increased with the grating disk size up to a maximum, before decreasing or saturating. The leftward shift of the peak of the spatial summation functions with higher grating spatial frequencies indicated that optimal size decreased with increasing spatial frequencies. Moreover, we observed a strong suppression of initial eye acceleration beyond the optimal sizes at low, but not high, spatial frequency where peak eye acceleration simply saturated with larger and larger stimuli.

**Figure 5.**
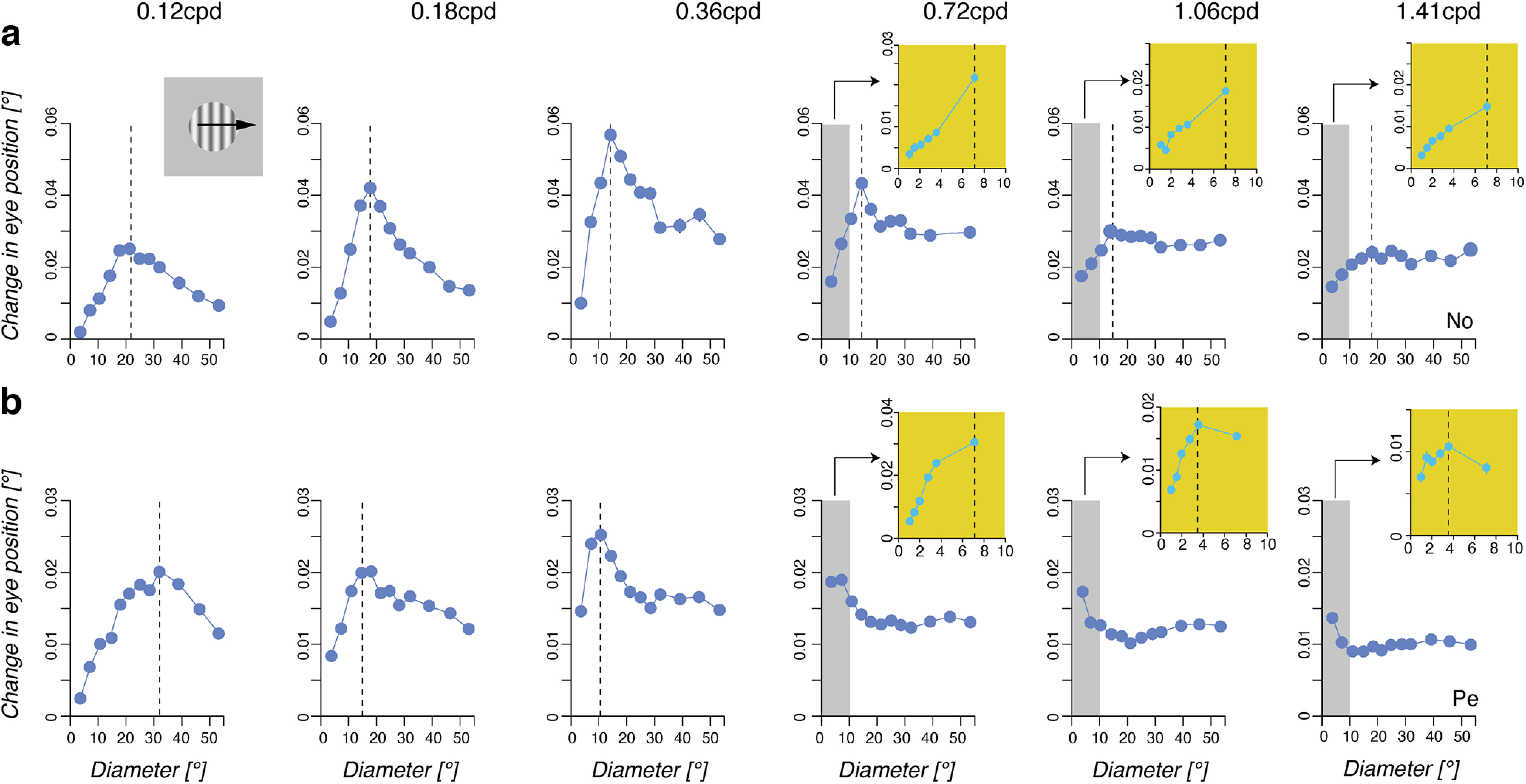
Mean stimulus spatial frequency shapes the spatial summation function. Early spatial summation functions are plotted for each of the six mean spatial frequencies (experiment 3). For the three highest spatial frequencies (0.72, 1.06, and 1.41 cpd), an additional experiment was run with a complementary set of smaller grating disk sizes. This experiment with smaller diameters maps more precisely the change in ocular following over the gray-shaded area of the original spatial summation function. Only data (mean ± SE, *n* > 150) for rightward grating movements are illustrated, as the results with the leftward direction were essentially the same. ***a***, ***b***, Monkey No (***a***) and Monkey Pe (***b***).

**Figure 6.**
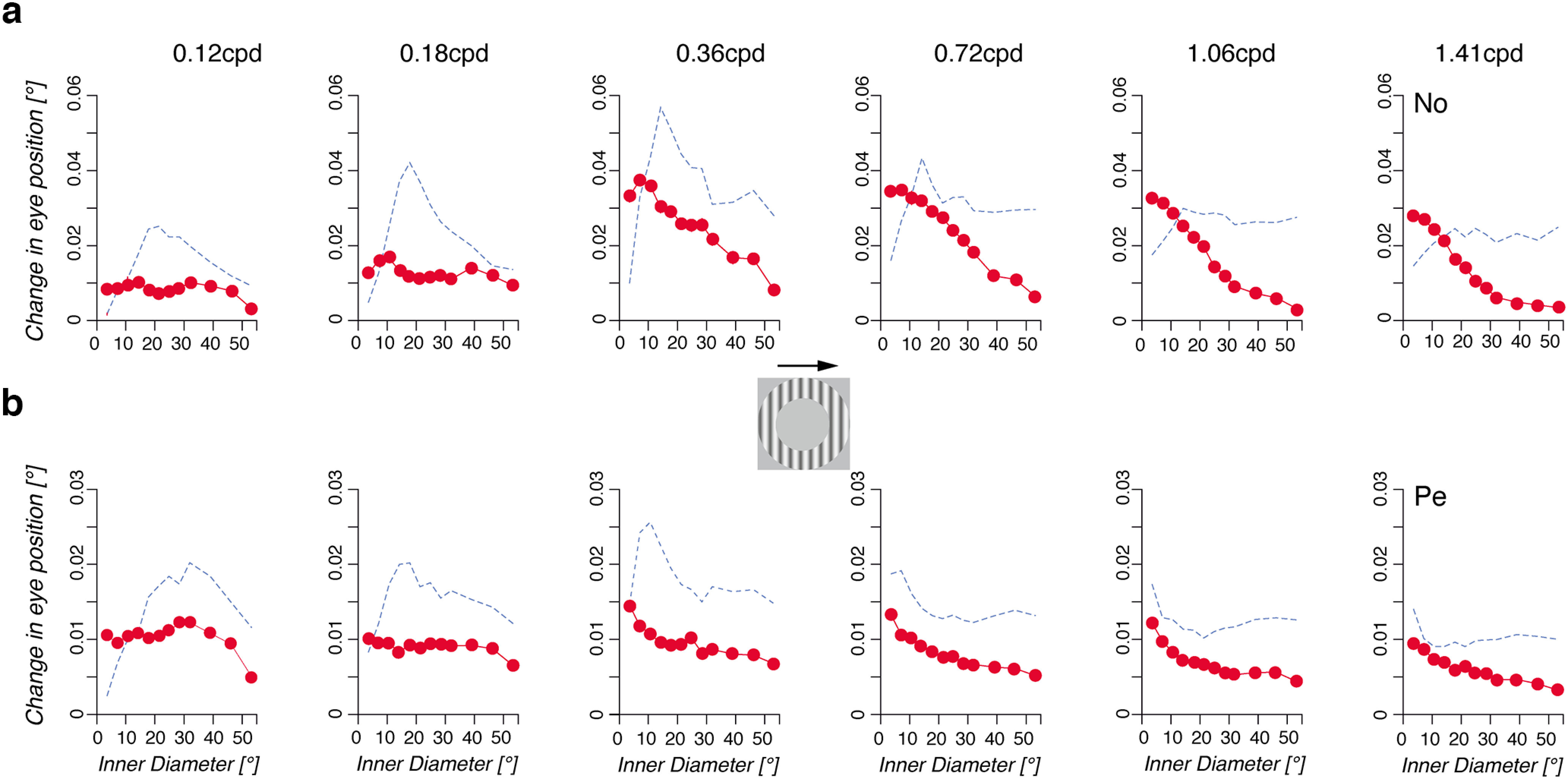
Contribution of peripheral motion changes with mean spatial frequency. Changes in early eye position (mean ± SE, n > 150 trials) are plotted against the diameter of the uniform circular patch covering the center of a large rightward motion stimulus (experiment 3). Each column corresponds to a given grating spatial frequency, ranging from 0.12 to 1.41 cpd. Dotted blue curves indicate the OFR spatial summation for the same mean spatial frequency, replotted from Figure 5. ***a***, ***b***, Monkey No (***a***) and Monkey Pe (***b***).

**Figure 7.**
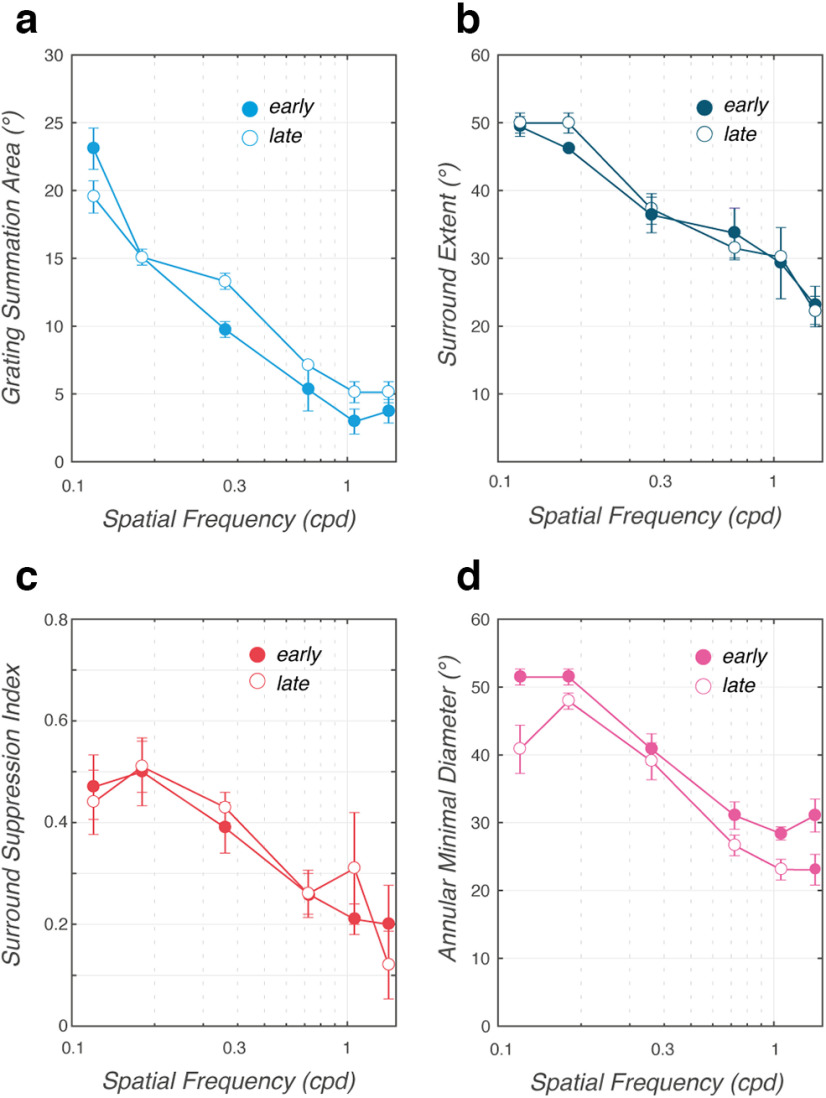
Relationships between spatial frequency and spatial summation. Mean (± SD; across animals and directions) characteristic indices of spatial summation are plotted against grating spatial frequency. Open and closed symbols illustrate the change in eye position over early and late time windows (65 -85 and 85 -105 ms, respectively). ***a***, GSA. ***b***, SExt. ***c***, SSI. ***d***, AMD. See Figure 2, *a* and *b*, for a description of these indices.

We measured the changes in eye position over two successive time windows, lasting 20 ms and starting at either 65 or 85 ms after grating motion onset. In Figure 5, we plot the second time window (85–105 ms), which shows a higher signal-to-noise ratio but still remains within the open-loop period of ocular responses. Mean changes in position (±SE; Fig. 5, bars are smaller than symbol) are plotted against stimulus diameter for each spatial frequency and monkeys No (Fig. 5*a*) and Pe (Fig. 5*b*), for the rightward grating motion direction. Similar results were obtained in the two monkeys with the leftward motion direction (see also Fig. 9*a*). Vertical dotted lines indicate the stimulus diameter for which the largest response was observed. From this value we extracted the grating summation area, as defined above. In monkey No, increasing the grating spatial frequency elicited a leftward shift of the dotted line (from >20° to <15°), indicating that optimal grating diameter decreased. The same trend was found in monkey Pe. However, for spatial frequencies of 1.06 and 1.41 cpd, response amplitudes decreased regularly over the whole range of tested diameters, making it impossible to estimate the optimal grating size. Therefore, we ran a control experiment with the two animals where ocular following responses to smaller grating diameters (from 1° to 7.1°) were mapped. Results are plotted within the color insets in Figure 5. One can see that for Monkey Pe the grating optimal size occurred for grating diameters <5° for the two highest spatial frequencies. We used this set of responses to recompute the GSA for the two grating directions. The mean GSA (±SD) across monkeys and grating motion directions are plotted in Figure 7*a* against grating spatial frequency. Open and closed symbols illustrate data at response onset (time window, 65–85 ms) and near the closing time of the oculomotor loop (85–105 ms), respectively. Increasing spatial frequency from 0.12 to 1.4 cpd (i.e., grating period varied from ∼8° to 0.7°) elicited a steep, exponential reduction of the GSA values from 19.5 ± 3.5° to 5.1 ± 2.3° and from 23.1 ± 4.6° to 3.7 ± 2.6° for the early and late 20 ms time windows, respectively (mean ± SD across monkeys and directions). Such a decrease was significant (0.12 vs 1.4 cpd conditions, *t*_(7)_ > 4.1, *p* < 0.01). A similar exponential decay was observed with SExt, indicating that the twofold ratio between center and surround spatial properties was maintained over the range of tested spatial frequencies (Fig. 6*b*). Overall, the mean (±SD) SExt decreased from 49.9 ± 4.4° to 22.1 ± 6.7° and from 49.4 ± 4.4° to 23.1 ± 8.4° for the early and late time windows, respectively (*t*_(7)_ > 3.3, *p* < 0.05). Across all spatial frequencies, the ratio between GSA and SExt was of 3.73 ± 2 and 0.7 ± 1.46 for early and late time windows (mean ± SD across monkeys, directions, and spatial frequencies). With both GSA and SExt, no significant differences were observed between early and late time windows.

The spatial summation functions illustrated in Figure 5, *a* and *b*, show some evidence that surround suppression observed with large stimulus diameters was stronger for low than for high spatial frequencies. Clearly, surround suppression was either very weak (monkey No) or nearly absent (monkey Pe) with the highest spatial frequencies. We computed the SSI for each condition and motion direction. Figure 6*c* plots the mean (±SD) SSI values against grating spatial frequency, for the two successive time windows. Increasing spatial frequency from 0.12 to 1.41 cpd resulted in a sharp, but not significant, decrease in SSI from 0.41 ± 0.2 to 0.12 ± 0.2 (early time window, open symbols: *t*_(7)_ = 1.95, *p* < 0.08) and from 0.47 ± 0.19 to 0.20 ± 0.2 (late time window, closed symbols: *t*_(6)_ = 1.62, *p* < 0.15). No differences were observed between early and late parts of ocular following responses. This result indicated that peripheral motion inputs had no, or very few, suppressive effects at high spatial frequency.

### The contribution of peripheral motion at different spatial frequencies

Figure 7 plots the earliest changes in eye position against the inner diameter of the ring stimulus, for a rightward grating motion. From left to right in Figure 7, grating spatial frequency increases from 0.12 to 1.41 cpd, as in Figure 5 For the lowest spatial frequencies (0.12 and 0.18 cpd), removing large portions of the central visual field did not affect ocular following until the inner diameter reached ∼40°. This indicates that, at low spatial frequencies, peripheral motion is sufficient to drive ocular following. However, further increasing grating spatial frequency dramatically changed this relationship. With higher spatial frequencies (1.06 and 1.41 cpd), weaker and weaker contributions of peripheral motion inputs are illustrated by the exponential decaying of response amplitude with inner diameter, down to almost zero for inner diameters >30°. Dotted lines replot the relationships between response amplitude and disk diameters (Fig. 5) to illustrate the fact that the reduction in surround suppression also corresponds to a reduced contribution of peripheral motion to ocular following.

AMD indices were computed for each direction and spatial frequency condition and for each monkey. Mean (±SD; across animals and motion directions) values are plotted against spatial frequency in Figure 6*d*, again for two different time windows. Increasing grating spatial frequency from 0.12 to 1.41 cpd resulted in a sharp, exponential decay of the mean AMD, from 40.8 ± 10.7° to 23.06 ± 6.8° (*t*_(7)_ = 3.19, *p* < 0.05) for the early time window, and from 51.4 ± 3.6 to 31.1 ± 7.2° for the late time window (*t*_(7)_ = 3.25, *p* < 0.05).

### Peripheral motion: grating or features motion?

**Figure 8.**
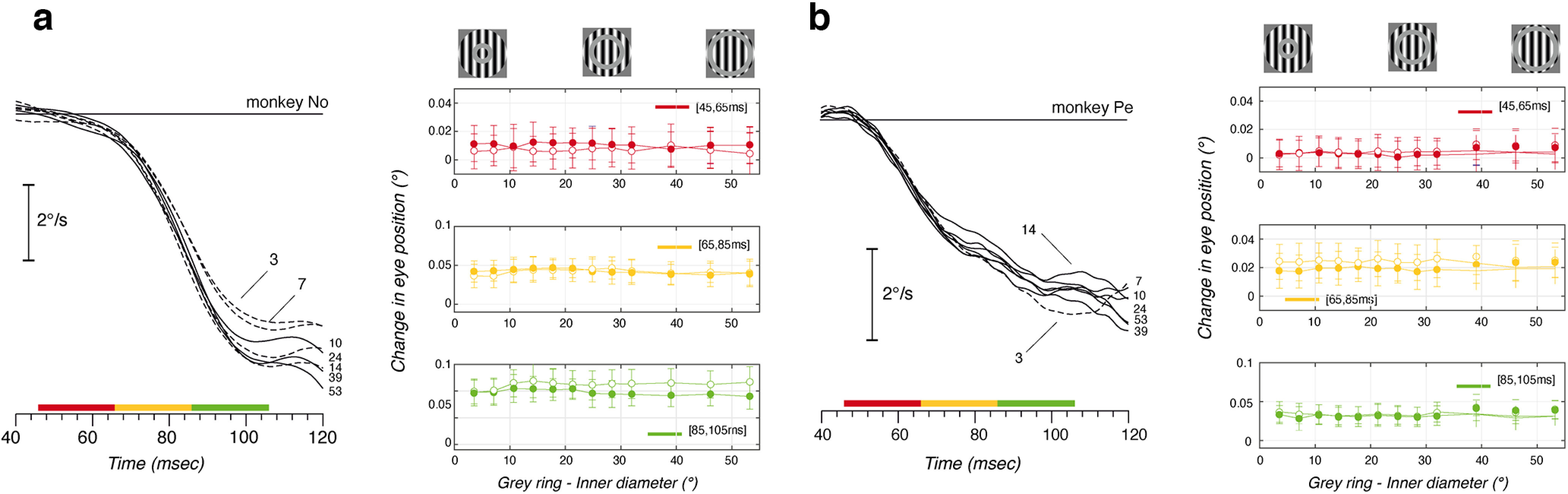
Surround inhibition is not because of line endings in the peripheral visual field. A masking ring (mean luminance) of 3.4° width and of increasing eccentricity is superimposed to a large grating patch (experiment 4). Such a mask introduces line-ending motion of an average direction identical to the moving grating. The left column shows mean eye velocity profiles, obtained with a masking uniform annulus of increasing eccentricity, placed over a leftward grating motion. The right column shows the change in eye position measured across three successive 20 ms time windows, plotted against the inner diameter of the mean luminance ring. Data are the mean ± SE (*n* > 100 trials). Closed and open symbols correspond to leftward and rightward grating motion directions, respectively. ***a***, ***b***, Monkey No (***a***) and (***b***) Monkey Pe.

In the two experiments reported above, we used a sharp edge circular aperture for both the disk and the ring stimuli. Two-dimensional (2D) features were thus created at the intersection between the grating luminance profile and the aperture, often called line endings. Since we used a circular aperture, the average motion direction of these line endings is equal to the grating motion direction. However, since several studies previously demonstrated the contribution of such 2D moving features to the late phase of ocular following (Masson et al., 2000; Barthélemy et al., 2010), we reasoned that the reduced eye velocity observed with large diameters could be explained by the increased eccentricity of these features, reducing their contribution to motion processing and tracking initiation. To test this hypothesis, we ran a control experiment (experiment 4) where a thin mean luminance annulus (thickness, 3.4°) of increasing eccentricity was superimposed over the largest grating disk. Thickness of the annulus minimized the changes in the overall motion stimulus surface. The eccentricity of line endings generated at the intersections between grating and ring apertures increased with the inner diameter of the ring. Those generated at the external borders of the grating disk remained of constant eccentricity. Ones can therefore probe the specific contribution of the ring-generated line endings on ocular following responses. Figure 8, *a* and *b*, illustrates the mean eye velocity of ocular following responses to different ring inner diameters. Note that moving gratings were presented at the individual, optimal spatial frequency (0.36 and 0.18 cpd for monkeys No and Pe, respectively; Fig. 5*a*,*b*). In both monkeys, the earliest phase of ocular following (i.e., <20 ms after response onset) was insensitive to the presence of 2D moving features. For larger annulus inner diameters, we found a weak but systematic modulation of eye velocity in monkey No, but not in monkey Pe.

To quantify the effects of line-ending eccentricity, we computed the changes in eye position over three successive 20 ms time windows, starting at 45, 65, and 85 ms after stimulus onset. In Figure 8, mean (±SE) response amplitudes (closed symbols: leftward motion; open symbols: rightward motion) were plotted in the right-hand columns, with three different colors indicating the different time windows. Overall, there was no significant modulation of ocular following amplitude when varying the inner diameter of the uniform annulus. For all conditions and time windows, linear regressions between response amplitudes and inner diameters were not significant (df = 10; *r*^2^ < 0.37; *p* > 0.2, across two monkeys). We found a weak modulation in response amplitude with inner diameter only on monkey No, for the leftward motion direction and only the last time window. When the inner diameter increased from 3.5° to 14.2°, response amplitude significantly increased (*t*_(128)_ = 2.08, *p* < 0.05) before saturating. This corresponds to the late change in eye velocities illustrated in Figure 8*a*. Overall, varying the eccentricity of the line endings generated at the intersection between grating and aperture luminance profiles had no effect on spatial summation.

### Modeling spatial summation for monkey ocular following

We have shown above that spatial summation of motion information for driving ocular following exhibits the following characteristics: response amplitude linearly increases with stimulus diameters before reaching a maximum value that signals the optimal stimulus size (experiment 1). Beyond such optimal size, response saturates at high spatial frequency but decreases at low spatial frequency corresponding to a surround suppression effect (experiment 3). The strength of surround suppression varies with spatial frequency and may be related to the relative contribution of the different spatial frequency channels to peripheral motion processing. Spatial properties of center–surround interactions slightly change over time. Last, spatial parameters of motion integration did not vary with grating speed or temporal frequency, at least when tested with a mid-range, optimal spatial frequency (0.36 cpd; experiment 2). Surround suppression was, however, stronger at higher speeds.

Integrating motion over a restricted, Gaussian-like portion of the visual field would result in a saturating spatial summation function, as observed with high spatial frequency. This is consistent with the observation that peripheral inputs contribute very little to ocular following. However, hypersaturation as observed with low spatial frequency cannot be explained with such a simple mechanism. When presented alone, peripheral inputs can drive ocular following. However, when presented together with a center grating that moves in the same direction, they seem to suppress the ocular following responses, depending on their spatial frequency. At a neuronal level, surround suppression mechanisms are classically described by either a DoG (Sceniak et al., 1999) or a ratio-of-Gaussians (RoG; Cavanaugh et al., 2002) model capturing inhibitory interactions between inputs located within different parts of the visual field. These models can also account for spatial summation of human motion perception (Tadin, 2015). Previous studies have suggested that a DoG model can well account for the spatial integration of visual motion information driving human ocular following responses (Barthélemy et al., 2006; Perrinet and Masson, 2007; Sheliga et al., 2013). For the sake of comparison between spatial summation in human and nonhuman primates, we defined a similar center–surround DoG model as follows:

$$f\left(x;\mu ,\sigma_{e},\sigma_{i},g_{e},g_{i}\right)=g_{e}\frac{1}{\sigma_{e}\sqrt{2\pi }}\text{exp}\left(\frac{-{\left(x-\mu \right)}^{2}}{2\sigma_{e}^{2}}\right)-g_{i}\frac{1}{\sigma_{i}\sqrt{2\pi }}\text{exp}\left(\frac{-{\left(x-\mu \right)}^{2}}{2\sigma_{i}^{2}}\right),$$

where $g_{\left(e,i\right)}$ and $\sigma_{\left(e,i\right)}$ are gain and SD parameters of the excitatory and inhibitory Gaussian functions, respectively.

**Figure 9.**
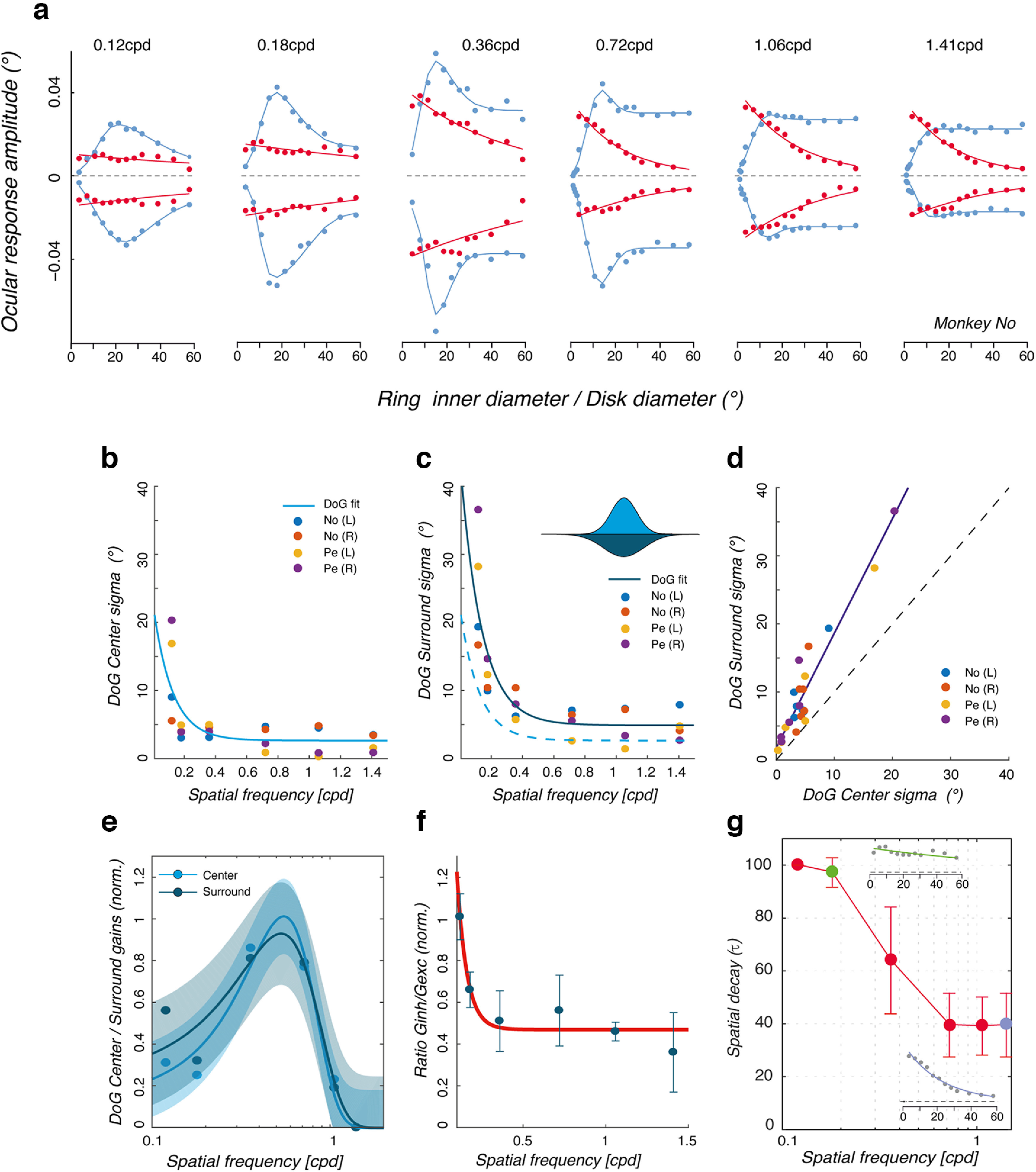
A difference-of-Gaussians model of OFR spatial summation. ***a***, A DoG model was fitted to the individual relationships between the change in eye position and stimulus size (blue dots) for each spatial frequency condition. A simple exponential decay function was fitted to the individual relationships between each change in eye position and the diameter of the central patch of mean luminance (red dots). Blue and red curves are best-fit functions, for Monkey Pe and for both rightward and leftward grating motions. Data are replotted from Figures 5 and 7. ***b***, ***c***, Best-fit σ_(_*_e_*_,_*_i_*_)_ parameters of center (***b***) and surround (***c***) Gaussian functions are plotted against grating spatial frequency, respectively. For both parameters, grating directions are plotted independently, for each monkey, together with the grand average values (continuous lines). In ***c***, the dashed line replots the exponential decay of σ_(_*_e_*_)_ from ***b*** to allow direct comparison. ***d***, The two sets of best-fit σ_(_*_e_*_,_*_i_*_)_ parameters are plotted against each other. Broken lines are the theoretical linear relationships with a gain of 1 or 2. ***e***, The gain *g*_(_*_e_*_,_*_i_*_)_ parameters of both central and surround Gaussians are plotted against grating spatial frequency, with confidence intervals. Continuous lines are best-fit Gaussian functions for either *g*_(_*_e_*_)_ or *g*_(_*_i_*_)_. ***f***, The ratio between excitatory and inhibitory gains (*g_i_*/*g_e_*), corresponding to the SSI computed above, is plotted against spatial frequency. ***g***, Mean values of the best-fit τ parameter, as a function of spatial frequency. The insets illustrate best-fit functions for rightward motion (monkey Pe), replotted from ***a***. In ***e–g***, data are the mean (±SD) across monkeys and directions.

Using the *erf* function, we fitted the DoG model to the spatial summation functions observed at different grating spatial frequencies. Notice that the fitted datasets were reconstructed by pooling the responses of the experiment 3 and the supplementary conditions using smaller diameters. For low spatial frequencies (0.12–0.36 cpd), we used the original dataset from experiment 3 (12 data points per direction) so that the data points for Monkey No are the same in Figures 5*a* and 9*a*. For high spatial frequencies (0.72–1.41 cpd), responses from the additional small (1–7.1°) and original (3.33–53.25°) diameters were aggregated after normalizing them relative to the common diameter (7.1°). Thus, there are now 16 data points (Fig. 9*a*, blue circles). Blue continuous lines in Figure 9*a* show the best-fitted DoG functions for the amplitude–diameter relationships in monkey No, for both rightward and leftward grating motion directions. Table 1 reports the mean (±SD; across monkeys and directions) values of the best-fit parameters.

**Table 1:** Mean (±SD) values of best-fit parameters of the DoG model

SF (cpd)	σ*_e_*	σ*_i_*	(σ*_i_ /*σ*_e_*)	*g_e_*	*g_i_*	*g_i_/g_e_*
0.12	12.96 ± 6.8	25.21 ± 0.04	2.15 ± 0.6	0.009 ± 0.005	0.008 ± 0.004	1.01 ± 0.22
0.18	3.98 ± 0.8	11.84 ± 2.14	3.03 ± 0.59	0.008 ± 0.005	0.005 ± 0.004	0.66 ± 0.17
0.36	4.18 ± 0.8	7.6 ± 2.1	1.85 ± 0.48	0.017 ± 0.003	0.009 ± 0.005	0.51 ± 0.29
0.72	3.04 ± 1.79	5.43 ± 1.98	2.10 ± 0.7	0.016 ± 0.01	0.16 ± 0.011	0.56 ± 0.34
1.06	2.62 ± 2.38	4.85 ± 2.93	2.98 ± 1.66	0.007 ± 0.006	0.004 ± 0.002	0.46 ± 0.09
1.41	2.36 ± 1.31	4.88 ± 2.2	2.36 ± 0.85	0.004 ± 0.003	0.001 ± 0.001	0.36 ± 0.38

The model captures the main properties of the ocular following spatial summation functions, and their dependencies on grating spatial frequency. In particular, beyond an optimal size in the range of 10–20° in diameter, the responses are suppressed at low, but not high, spatial frequencies. Data for each spatial frequency were fitted independently for each monkey and each motion direction to estimate how the spatial frequency of the grating modulates the different best-fit parameters. As a control, we fit the same functions to all spatial frequency data simultaneously, without any improvement. Figure 9, *b* and *c*, plots the individual best-fit values for the DoG center (σ*_e_*) and surround (σ*_i_*) parameters. Both parameters decrease with spatial frequency, and continuous lines plot the best-fit exponential decay to all individual data. Still, when plotted one against the other (Fig. 9*d*), their ratio remains constant across the spatial frequency range with a mean ± SD value of 2.41 ± 0.92 (across monkeys, directions, and spatial frequencies). There was a significant linear relationship between σ*_e_* and σ*_i_* with a slope of ∼2 (slope = 1.7, intercept = 1.78, *r*^2^ = 0.9, df = 22, *p* < 0.05). The ratio between best-fit σ*_i_* and σ*_e_* parameters was constant across spatial frequencies. For instance mean (±SD) values of the σ*_i_/*σ*_e_* ratio were of 2.15 ± 0.6 and 2.36 ± 0.84 for the lowest and highest frequencies, respectively (*t* test, *t*_(7)_ = 0.9, NS).

Excitatory and inhibitory gain parameters both vary with spatial frequency, peaking for mid-range frequencies (0.36–0.72 cpd; Table 1). To best estimate these spatial frequency tuning, we normalized (between 0 and 1) across spatial frequencies the best-fit values of the *g_e_* and *g_i_* parameters of the DoG model. When plotted against grating spatial frequency, both normalized parameters exhibit an inverted U-shaped spatial frequency tuning function, centered on frequencies between 0.4 and 0.5 cpd (Fig. 9*e*). These spatial frequency tunings were best fitted by two Gaussian functions in linear space and plotted in log space in Figure 9*e*. SDs of the Gaussian tuning ( $\sigma_{\left(sf\right)}$) for the excitatory and inhibitory functions were 0.31 and 0.26 cpd, peaking at 0.53 and 0.55 cpd, respectively (Fig. 9*e*, continuous lines), strongly suggesting that center and surround mechanisms are similarly tuned for spatial frequencies.

Modeling spatial summation for ocular following allows estimating the relative contribution of central and peripheral motion. Figure 9*f* plots the relative weight of *g_e_* and *g_i_* (i.e., the ratio *g_i_/g_e_*) as a function of spatial frequency. As grating frequency increased from 0.12 to 1.41 cpd, *g_i_* was halved and therefore the ratio *gi/g_e_* decreases from ∼1 to ∼0.4 (Table 1). This result is consistent with the decrease of the model-free SSI that was reported above when spatial frequency increased (Fig. 5*c*). The DoG model shows that inhibitory surround inputs are weaker at high spatial frequency and that spatial summation is mostly determined by the integration of central motion inputs. Ring-like stimuli were indeed designed to probe such a peripheral contribution (Fig. 6). The relationship between ocular following amplitude and ring inner diameter was modeled by the following exponential decay function:

$$f\left(x;g_{r},\tau ,r_{0}\right)=g_{r}e^{-x/\tau }+r_{0},$$

where *g* and *r*_0_ are gain and offset parameters and τ is the decay size constant (in degrees). Maximal amplitude was limited to 100°. At very low spatial frequency, τ was large (97.2 ± 5.5° at SF = 0.18 cpd), indicating that the peripheral motion contribution was weakly dependent on its eccentricity (Fig. 9*g*). By contrast, at the higher spatial frequencies, τ was reduced to ∼40° (e.g., 39.5 ± 12° at SF = 1.41 cpd), indicating a weaker drive of motion signal presented beyond 10° of eccentricity (i.e., a ring with an inner diameter of ∼20°; Fig. 7). This result is consistent with the reduction from 44° to ∼12° that was reported above with the model-free AMD index when spatial frequency increased from 0.12 to 1.41 cpd (Fig. 6*d*). Thus, there was a weak contribution of higher spatial frequencies when presented beyond 10° of eccentricity for either driving or suppressing ocular following responses.

## Discussion

Here, we document the dynamics of center–surround interactions shaping the early phase of ocular following eye movements in macaque monkeys. These properties reflect many characteristics of context-dependent motion integration in macaque area MT as reported by others (for a recent review of quantitative data, see Vanni et al., 2020).

### A center–surround mechanism of motion integration

It has long been thought that reflexive tracking eye movements, also called optokinetic responses, are driven by the en masse motion of the visual field (Miles and Kawano, 1987). However, several studies demonstrated that full-field motion is not the optimal stimulus for both human optokinetic eye movements (Ter Braak, 1957; Howard and Ohmi, 1984; Abadi et al., 2005) and primate ocular following responses (Miles et al., 1986; Gellman et al., 1990). When presented with large (40° across) central motion stimuli, macaque ocular following responses are larger when images in the surround move in the opposite rather than in the same direction of the center. By contrast, a stationary surround produces a weak suppression (Miles et al., 1986). Similar center–surround direction-selective modulations were observed with human ocular following (Gellman et al., 1990). Moreover, these spatial interactions between opposite motions are binocular disparity selective (Busettini et al., 1996; Masson et al., 2001). These seminal results opened the door to investigating the exact properties of the center–surround mechanisms underlying spatial integration of motion information. In humans, Barthélemy et al. (2006) first documented that initial eye velocity linearly increases with grating diameters up to 20° and then saturated. Lower contrast gratings yield to larger optimal central diameters. Some evidence was found for a decrease in response amplitude with larger stimulus sizes, but surround modulation was weak and not reliable across participants. Using a different stimulus configuration, Sheliga et al. (2015) reported a similar optimal integration zone, although suppression was stronger. Here, we report for the first time in monkeys the existence of strong surround suppression for ocular following. At optimal spatial frequency (0.36 cpd), the earliest eye velocity was halved when comparing largest (∼60°) with optimal (∼16°) grating diameters. The resulting spatial summation function for a circular grating patch exhibits the classic nonmonotonic shape where motion signals are first linearly integrated before response amplitude saturates when stimulus size reaches an optimum that is much smaller (diameter, ∼20°) than usually thought for a monkey optokinetic eye movements. Beyond the optimal integration zone, peripheral motion signals suppress ocular responses. Such suppression saturates when stimulus diameter reaches nearly twice the size of the optimal integration zone. We compared ocular following to disk and ring-like stimuli to titrate the contribution of peripheral motion signals. First, ocular following linearly (in log-scale) decreases when the inner diameter of the ring increases, reaching 5% of the maximum response when motion signals were presented at eccentricity >20°. Second, with our conditions, it is noticeable that two of our ring and disk stimuli had a nearly identical stimulus surface, but located at different eccentricities: the ring surface extended between 26° and 30° eccentricity, while the disk surface covered the central 14°. Responses to the ring were only ∼25% of those obtained with the disk. Thus, peripheral motion can only weakly drive ocular following but can instead strongly suppress the center-driven responses.

Inspired by the work of Cavanaugh et al. (2002), we first extracted model-free parameters to describe spatial summation. For a 0.36 cpd grating, we found a strong consistency of the values of these characteristics across monkeys and motion directions: the central 15° drives ocular responses, surrounded by a strong suppressive surround of ∼10° width. Peripheral motion signals at eccentricity >20° can hardly drive or modulate ocular following responses. These values are consistent with previous reports using different stimuli in both humans (Gellman et al., 1990; Sheliga et al., 2008a,b 2013) and macaques (Miles et al., 1986). Next, we fitted a DoG model to obtain model-based parameters of the center–surround mechanisms. Results were again remarkably consistent. Best-fit SDs [σ_(_*_e_*_,_*_i_*_)_] of the excitatory and inhibitory components were ∼4° and 8°, such that the full-width at half-maximum values (defined as 2.35; σ_(_*_e_*_,_*_i_*_)_) were ∼10° and ∼18°, respectively. Interestingly, the ratio between center and surround diameters was ∼2 and remained stable with respect to all other parameters investigated such as spatial and temporal frequencies or speed. Such best-fit center SD ( $\sigma_{e}$) corresponds to approximately three cycles of the moving grating.

### Mapping spatial integration: local versus extended stimuli

Spatial properties of the receptive fields of neurons have been mapped using either extended inputs of varying sizes (De Angelis et al., 1994; Sceniak et al., 1999; Cavanaugh et al., 2002) or local inputs of varying locations (Barlow, 1953). The same dual approach was used for ocular following. Some groups used expanding patches of drifting gratings to estimate the region of summation, in both humans (Gellman et al., 1990; Barthélemy et al., 2006) and macaques (Miles et al., 1986). Present results are highly consistent with these seminal studies, in both estimating the optimal size (diameter, 10–15°), the reduced contribution of peripheral motion and the spatial properties of surround suppression. Sheliga et al. (2008a,b, 2012) used a different approach, manipulating the number of nonadjacent thin rectangular stripes. Their seminal observation was that a very thin, horizontal stripe (1× 47°) of moving grating can still elicit ocular following at the typical short latencies (∼90 ms) in humans (Sheliga et al., 2008a,b). Initial response amplitude increases with strip heights up to ∼10°, at optimal spatial frequency (Sheliga et al., 2012), are consistent with the optimal size reported by Barthélemy et al. (2006). Their stimulus allowed variation of the separation between pairs of stripes. When it was >10° (i.e., the eccentricity of each strip was >5°), responses decreased. When increasing the number of thin stripes presented within the central 10°, response amplitude is best predicted as a spatial, weighted averaging of local inputs (Sheliga et al., 2012). This result is consistent with the linear relationship between response amplitude and stimulus size, within this range, as reported both here in macaques and in humans (Barthélemy et al., 2006). It is also consistent with both spatial summation and surround suppression observed when using multiple Gabor patches (Barthélemy et al., 2006).

Here, we document in macaques a strong surround inhibition with large stimuli. At optimal spatial frequency, human ocular following to very large patterns is smaller than with optimal ones (Barthélemy et al., 2006; Sheliga et al., 2008a, 2012). However, the suppression is much smaller in humans (∼15–20%) than in macaques (∼50%) and gradually builds up such that it becomes evident only in the late part of the response. Interestingly, the strength of surround suppression reported herein is similar to that reported in macaque for motion direction discrimination using gratings in a perceptual task (Liu et al., 2016). Barthélemy et al. (2006) reported that surround suppression is direction and orientation selective and dependent on surround contrast, suggesting a divisive normalization mechanism similar to what has been reported at neuronal and perceptual levels (for review, see Carandini, 2004; Carandini and Heeger, 2011). Using their pattern of motion stripes, Miles et al. (1986) identified the contributions of local and global normalization mechanisms (Sheliga et al., 2008a,b). Sheliga et al. (2012, 2015) further demonstrated that global normalization is orientation and spatial frequency dependent, arguing for a cortical origin. Overall, multiple local motion inputs and large patterns provided consistent information about the spatial properties of motion integration for ocular following, as we have reported in humans (Barthélemy et al., 2006). Such a comparison would still be interesting to perform in monkeys as it can be more directly related to physiological properties of cortical motion integration. However, we did not test extensively a normalization model that could be implemented as an RoG rather than a DoG, as it would require exploration of other grating properties such as relative orientation or contrast between center and surround (Cavanaugh et al., 2002; for modeling ocular following dynamics, see Perrinet and Masson, 2007).

### Spatial frequency tuning of motion integration

Miles et al. (1986) reported that the optimal spatial frequency range for driving ocular following is ∼0.01–1 cpd in macaque monkeys (Miles et al., 1986; Miura et al., 2006). When considering early response amplitude, spatial frequency tuning is Gaussian shaped in log space, peaking at ∼0.3 cpd (Miura et al., 2006). We report that over this spatial frequency range, spatial summation exhibits gradual changes in both optimal integration area and the strength of surround suppression. First, the optimal integration zone shrinks with higher spatial frequencies, as demonstrated by the relationship between grating spatial frequency and both the (model-free) GSA and the DoG-fitted σ*_e_* parameters. Estimating the Gaussian tuning functions of the DoG excitatory gain ( $g_{e}$) showed that the gain peaked at low frequencies (∼0.5 cpd) with an SD ( $\sigma_{sf}$) of ∼0.3 log units, values that are very similar to those reported earlier in monkeys (Miles et al., 1986; Miura et al., 2006).

Using disk-like stimuli, we report that high spatial frequency can hardly drive, and modulate, ocular following when located in the periphery. Moreover, over our range of spatial frequencies (∼4 octaves), σ*_e_* and σ*_i_* decrease fourfold, but their ratio remains constant at ∼2. Moreover, both ratio (*g_i_*/*g_e_*) and the SSI parameters are strongly reduced, indicating a weaker surround suppression at higher spatial frequencies. These results are consistent with previous studies in human ocular following (Quaia et al., 2012; Sheliga et al., 2012). They are also consistent with the shift in contrast sensitivity toward lower spatial frequency with eccentricity, as reported both in humans (Rovamo et al., 1978) and monkeys (De Valois et al., 1974). It can be explained by the scarcity of high spatial frequency channels at large eccentricities, while low spatial frequency channels are distributed more evenly across the visual field (De Valois et al., 1982; but see De Valois and De Valois, 1988). Our study provides evidence for a direct and quantitative link among spatial summation, surround suppression, visual eccentricity, and spatial frequency tuning in the macaque visual system.

### The spatial and temporal properties of macaque bRF

Masson and Perrinet (2012) proposed that motion integration mechanisms driving ocular following are best condensed as a “behavioral receptive field” emulating several linear and nonlinear computations classically described for sensory neurons. Previous work in both humans and macaques have detailed how ocular following contrast response function is best described by a divisive normalization mechanism (Sheliga et al., 2005, 2008a,b; Barthélemy et al., 2006, 2008, 2010). The same computation probably underlies spatial interactions between competing motions (Barthélemy et al., 2006; Perrinet and Masson, 2007; Sheliga et al., 2008a,b). The present study details the spatial organization of such a monkey bRF. We found that motion inputs within the central 10° contribute more strongly in driving ocular tracking initiation than peripheral motion. Such a central, driving zone is dependent on the spatial frequency content of the visual motion inputs, which is larger for lower frequencies. At higher spatial frequency, the optimal diameter can be narrowed down to ∼5°, confirming that a small motion stimulus is sufficient to drive ocular following at ultrashort latency in monkeys, as reported in humans (Sheliga et al., 2008a, 2015). Moreover, at a given spatial frequency, spatial summation is independent of temporal frequency and speed. This implies that spatial summation is primarily constrained by the inverse relationship existing between spatial frequency tuning and eccentricity, as found at the earliest stages of the primate visual motion pathway, from retina to cortical area MT in both humans (Henriksson et al., 2008; Aghajari et al., 2020) and monkeys (De Valois et al., 1982; for review of quantitative data, see Vanni et al., 2020).

Central inputs are, however, strongly suppressed by peripheral motion signals of the same spatial frequency, contrast, and direction. Again, center–surround interactions are mostly insensitive to temporal frequency and speed, suggesting that the main factor is the spatial frequency tuning of the surround. Over a broad range of spatial frequencies, the relative sizes of central and surround areas remain nearly constant, with a ratio of ∼2. The surround suppressive effects are larger in monkeys than in humans (Barthélemy et al., 2006) when using the same stimulus configuration. In humans, the bRF exhibits a consistent orientation tuning of surround suppression, with iso-oriented inputs being more suppressive than cross-oriented ones (Barthélemy et al., 2006). We did not investigate here surround orientation or contrast. However, it is known that the contrast response function of ocular following is modulated by surround motion, regardless of its orientation in monkeys (Reynaud et al., 2007). This would suggest that surround suppression acts as a global normalization mechanism. A systematic comparison between human and macaque ocular following would allow the deciphering of the specific properties of contextual modulation for visual motion integration and comparison of the underpinning physiological properties of human and monkey motion pathways.

Another key difference between spatial summation and surround suppression in humans and monkeys is related to its timing. A property of ocular following is its ultrashort latency in both humans (∼90 ms) and macaques (∼55 ms). In monkeys, tracking onset is locked in a timely way to the population neuronal response onsets in cortical areas MT and MST (∼45 ms; Kawano, 1999) and the cerebellar oculomotor structure (∼50 ms; for review, see Kawano et al., 1994; Masson and Perrinet, 2012). It is therefore possible to track the temporal dynamics of motion integration, nearly millisecond by millisecond, in both humans (Masson et al., 2000; Masson and Castet, 2002; Barthélemy et al., 2008) and monkeys (Barthélemy et al., 2010), and relate it to neural dynamics (Masson and Perrinet, 2012). In humans, Barthélemy et al. (2006) originally reported that surround inputs already suppress ocular following at response onset. We observed the same ultrafast dynamics in macaques. At high contrast, spatial summation and surround suppression operate with the same neuronal delays, suggesting that such center–surround interactions reflect the properties of a neuronal population integrating inputs through a fast feedforward–feedback mechanism (Angelucci et al., 2017). However, in humans, contrast normalization as well as orientation tuning of surround suppression build up over ∼40 ms (Barthélemy et al., 2006), consistent with the delayed local, tuned suppression reported by others (Sheliga et al., 2008a,b). In the present study, we did not vary surround orientation but such a local and slower suppression mechanism was probably unveiled when introducing a small mean luminance ring generating local motion features. We report that such features barely suppress ocular following, and when they do it in one monkey, their impact is delayed by ∼30 ms, regardless of their eccentricities. These two series of results draw the temporal structure of the bRF where global and local mechanisms may be driven by different motion mechanisms with different timing. It is interesting to note that such dynamics are similar to those reported for 2D motion integration in both human and monkey ocular following (Masson and Perrinet, 2012), which is known to be strongly dependent on center–surround mechanisms in areas V1 and MT (Tsui et al., 2010). Perrinet and Masson (2007) proposed a unified computational framework, based on dynamic inference and able to implement altogether gain control, spatial summation, surround suppression, and 2D motion integration based on center–surround interactions at behavioral levels. To explain the dynamics, one hypothesis is that local and global suppression mechanisms act at different scales, and thus at a different steps along the visual motion hierarchy (Angelucci et al., 2017).

### Which neural mechanisms?

In monkeys, ocular following responses are driven by signals flowing from cortical areas MT and MST to the tracking oculomotor system via the dorsolateral pontine nucleus (Kawano et al., 1994; Kawano et al., 2000). Spatiotemporal tuning of ocular following is best understood as a combination of the population tunings of these two cortical areas (Miura et al., 2014). It is, however, difficult to isolate the contributions to ocular following from one or another area (Masson and Perrinet, 2012). For instance, speed tuning of macaque ocular following is similar to that of MST neurons and similarly modulated by binocular disparity (Kawano et al., 2000; Masson and Perrinet, 2012). Overall, the fact that MT surround suppression is highly dependent on several stimulus properties such as orientation, contrast, and signal-to-noise ratio (Born and Bradley, 2005) argues for an adaptive normalization mechanism tuned for selective motion integration (Born et al., 2000; Huang et al., 2007). Many properties of ocular following strongly suggest a key role of area MT. First, the temporal dynamics of macaque responses to plaid patterns (Barthélemy et al., 2010) mirrors that of area MT neurons (Smith et al., 2005). Second, the sizes of optimal integration for ocular following (i.e., diameter between 5° and 15°, depending on spatial frequency) matches the range of receptive field sizes in area MT (Raiguel et al., 1995; Born, 2000), whereas area MST neurons have much larger receptive fields (Miura et al., 2014). A majority of MT neurons have receptive fields with antagonistic surrounds, yielding to nonmonotonic spatial summation functions with a reduction in neuronal firing with stimuli larger than optimal (Allman et al., 1985; Born and Bradley, 2005; Vanni et al., 2020). Interestingly, the ratio between center and surround sizes for ocular following falls within the mean range (approximately two to three times) reported for MT receptive fields (Raiguel et al., 1995). Moreover, the strength of surround suppression (∼50% at high contrast) is also very similar between initial tracking and MT neuronal activity at the population level (Pack et al., 2005). Overall, spatial summation properties of bRF could be set by those of direction-selective cells in area MT, before their nonlinear integration forms MST receptive fields (Mineault et al., 2012). A complete depiction of the dynamic properties of center–surround interactions for monkey ocular following will be very helpful in sorting the contribution of different areas to a simple behavioral system.

The sources of MT surrounds remain unclear. One possibility is that the surrounds are already present in the inputs to MT. However, the sizes of the MT surrounds, and their complexity make this an unlikely explanation (Born and Bradley, 2005). Rather, MT surround properties may reflect either feedback interactions from higher areas such as MST or horizontal connections within MT itself (Malach et al., 1997). Grabbing altogether the properties of ocular following (Barthélemy et al., 2006; Sheliga et al., 2008a,b, 2012, 2015) allows the proposal of an alternative framework where both local and global interactions coexist. A local normalization mechanism would be responsible for gain control. It is tuned for orientation and contrast and could be implemented through nonlinear, lateral interactions at the level of area V1. This could explain the slow buildup of gain control, over cortical distances covering a few degrees of visual angle (Reynaud et al., 2012). The global normalization mechanism, untuned for orientation and direction and operating over much larger distances, would result from either the convergence of MT inputs or lateral interactions within area MT. The hierarchical model of motion integration that we proposed earlier for ocular following (Perrinet and Masson, 2007; Barthélemy et al., 2008) and visual motion perception (Tlapale et al., 2010; Perrinet and Masson, 2012) combines feedforward and lateral interactions with different spatial and temporal scales to simulate the dynamics of motion integration. Our new behavioral data, obtained in monkeys will ease the definition of a more complete model integrating biological constraints from macaque physiology.

### Conclusions

We show here that it is possible to map the spatial properties of motion integration in macaque monkeys with an unprecedented resolution, linking behavioral dynamics with neural properties of visual motion computation at single cells and population levels. Properties of the spatial integration mechanisms for either voluntary smooth pursuit eye movements (Heinen and Watamaniuk, 1998; Mukherjee et al., 2017; Debono et al., 2010) or perception (Tadin, 2015) have been explored in humans with consistent results in terms of center–surround interactions. For instance, Debono et al. (2010) defined an “oculoceptive” field driving human steady-state pursuit with spatial extent (<10°) that matches the present results. Moreover, for voluntary pursuit, these spatial properties are often modulated by contextual effects such as the structure (Masson et al., 1995; Spering and Gegenfurtner, 2007) and richness of the visual scene (Goettker et al., 2020), feature motion saliency, and spatial attention (for review, see Souto and Kerzel, 2021). Simple tracking responses such as ocular following can efficiently complement these approaches, with the opportunity to map with high spatial and temporal resolution the different preattentive mechanisms operating at different spatial and temporal scales and link them to basic physiological mechanisms along the primate motion pathway.

## Synthesis

Reviewing Editor: David Schoppik, New York University - Langone Medical Center Decisions are customarily a result of the Reviewing Editor and the peer reviewers coming together and discussing their recommendations until a consensus is reached. When revisions are invited, a fact-based synthesis statement explaining their decision and outlining what is needed to prepare a revision will be listed below. The following reviewer(s) agreed to reveal their identity: Boris Sheliga.

Briefly, the questions posed in the manuscript are interesting, the methods appropriate, and we will be happy to see a resubmission. However, we all noted that to be suitable for publication in eNeuro, the manuscript needs a considerable overhaul. I’ve included the Reviewers’ detailed comments.

One final suggestion is to reconsider the way you’re treating past work addressing questions in the smooth pursuit literature. For example, “But spatial integration for pursuit is for instance also dependent upon attentional modulations (Schütz et al., 2015)” Your references don’t include the Schütz paper, which presumably is 10.3389/fnhum.2015.00566 and doesn’t really support the idea. Further, ocular following can be similarly influenced cf 10.1152/jn.00810.2013 .

Comments (R1)

The paper asks interesting questions and presents results of a series of experiments designed to tackle those. Unfortunately, the paper is very poorly written. Many statements in the text of the manuscript are not supported by the data shown in the Figures. Several Figures themselves are very hard to interpret. The description of the methods is sometimes confusing. Certain aspects of the data are not acknowledged in the text, though I consider them to be important for understanding and interpreting the results.

Below I will give just several examples, which illustrate shortcomings in the manuscript, in no particular order.

P9. “For the earliest ocular following, the mean ({plus minus}SD across monkeys and directions) GSA was of 15.9{plus minus}2.1{degree sign}. The latest time window yielded very similar results (11.5{plus minus}1.8{degree sign}).” In Figure 2C not a single value of GSA exceeds 10{degree sign}. So I do not understand how such mean values could surface. In addition, the ordinate axis in Figure 2C looks very strange: it’s not a linear one, but it does not look like a logarithmic one either. Figure 2D ordinate is labeled “Surround Diameter” whereas Sext values are plotted. Actually, Figure 2D is redundant: the conclusion in the text that it illustrates should surface from the inspection of Figure 2C. There is no description in the text as to how GSA parameters (Sext, AMD as well) were calculated: was it an interpolation between two data points abutting 95% level, or were they based on some fitting, and the GSA value was assigned when the fit hits 95% level? There is no way to know.

Figure 1a,b left column: there is a prominent deflection in eye movement velocity traces at ∼30 ms latency. What is it? There is no mention of this [troubling] feature of the data in the text.

It is stated that Figure 9A replots rightward motion data of monkey Pe from Figures 4 and 5. First, the data appear to come from monkey No, not Pe, but more importantly, response amplitudes are ∼3 smaller in Figure 9A than they are in Figure 5.

P18 “...parameters of motion integration did not vary with grating speed or temporal frequency...” Compare to P12 “Higher speed (45{degree sign}/s) elicited larger suppression, when compared to lower speed...”

P16 “Increasing grating spatial frequency resulted in a sharp, exponential decay of the mean AMD, from 44.4{plus minus}3.6 to 12.4{plus minus}2.5{degree sign}...” Even by eye inspection of Figure 7, I don’t see AMD values dropping to 12.4{degree sign} at high spatial frequencies.

P14 “However, for spatial frequencies of 1.06 and 1.41cpd, response amplitudes decreased regularly over the whole range of tested diameters...” Not true, see 2 right columns of Figure 5.

The results of the last control experiment (Figure 8) were quite different for monkeys No and Pe. They also do not add anything substantial to the story. In my view, this experiment can be dropped from the paper. In addition, the description of this experiment in Methods (P6; “In the last two experiments...” is very confusing.

P9 “We defined the Surround Extent (SExt) as the diameter of the smallest stimulus for which initial eye velocity reached 5% of its asymptotic value observed for the largest gratings” The authors certainly meant to say “within 5% of its asymptotic value”.

P9 “The Annulus Minimum Diameter (AMD) is the first inner diameter for which response amplitude reached 5% of the smallest response...” The authors certainly meant to say “...of the largest response...”.

Figure 2 legend “As larger and larger central portions of stimulus were removed, responses amplitude regularly decreased, following a nearly exponential decay.” Only one dependence out of 4 in Figure 1C can possibly be characterized this way.

P5 “As speed, but not temporal frequency, is known to impact OFRs amplitude...” I think, the opposite is true.

P5 “...6 different spatial frequencies were used (0.12, 0.18, 0.36, 0.72, 1.06 and 1.41cpd) spanning about 2.4 octaves...” I think, it’s 3.6 octaves

P6 “To eliminate possible contamination of the eye velocity profiles from spurious slow ocular drifts, all data shown here have a fixation-only (blank stimulus) condition subtracted.” I think, it’s a rather problematic choice. Right-left difference traces could be a better solution.

Comments (R2)

In this well-conceived set of studies, the authors attempt to further characterize the ocular following response in NHPs using manipulations of stimulus diameter (spatial summation) and spatial frequency. In addition, the authors largely rule out the role of line-endings (stimulus features) in driving ocular following, particularly during the early initiation of the following response. Finally, the authors fit a difference of Gaussians model to their psychophysical data - the standard deviations of the center and surround Gaussians tended to agree with the authors’ model free estimates inhibition across spatial frequency.

In general, this is a thorough and well-conducted study. I also appreciate that the authors, where possible, chose to show the entire eye velocity timeseries traces rather than simply averaging. These behavioral results are also incredibly valuable from the standpoint of understanding early visual processing (MT/MST), and have important implications for understanding neuron responses to similar stimuli in these areas.

Major comments:

- The prose is somewhat difficult to follow. Much of this difficulty comes from the lack of a consistent narrative that links the experiments together, or even motivates each experimental observation/analysis. At the very least, the conclusions described in the paragraph on page 17 (beginning the section “Modeling spatial summation for monkey ocular following”) should be incorporated into the preceding results sections. In addition, motivating why the particular model-free measures are used should be included in the manuscript. While I understand that these parameters are taken from other research, it is still useful to link these parameters their potential mechanistic underpinnings. For instance, when introducing SExt (page 9), it would be useful to note that this is a metric which is linked to the potential width of surround suppression. In short, this manuscript would significant benefit from careful revision to the text with a clear emphasis on the motivation and conclusions of each experimental observation.

- The current DOG model adds little beyond confirming the authors’ prior model-free observations. The major issue, as I see it, is that the authors re-fit the model for each of the spatial frequencies independently. It is clear from the form of the DOG that such a model would be able to fit all of the authors’ experimental conditions/curves in the top panels of Figure 9A. Yet, that exercise in model fitting does not really provide any novel predictions nor insights into the previously described data. A far more powerful model would simultaneously fit the data across spatial frequencies (e.g., potentially using divisive normalization). Such a model may be able to provide predictions about potential future behavioral or neurophysiological experiments. As it stands, I would lean towards removing the model from the manuscript unless the authors can provide an alternate form/improve the current model’s predictive power.

Minor comments:

- Methods section (page 5): First use of the abbreviation “cpd” is missing the “d”. Currently written in the text as “0.36cp”.

- Method section: “Marquard-Lindberg” algorithm used to fit the model? Is this supposed to be Levenberg-Marquardt?

- Figure 3 - It is unclear why both right and left are included in this figure when elsewhere in the manuscript only rightwards motion directions.

- Figure 4 - The insets seem unnecessary. All other plots show change in eye position (which is shown for both monkeys in Figure 5). I am unclear why peak acceleration is plotted here, especially considering that it largely mirrors the results in Figure 5.
